# Identifying Molecular Properties of Ataxin-2 Inhibitors for Spinocerebellar Ataxia Type 2 Utilizing High-Throughput Screening and Machine Learning

**DOI:** 10.3390/biology14050522

**Published:** 2025-05-08

**Authors:** Smita Sahay, Jingran Wen, Daniel R. Scoles, Anton Simeonov, Thomas S. Dexheimer, Ajit Jadhav, Stephen C. Kales, Hongmao Sun, Stefan M. Pulst, Julio C. Facelli, David E. Jones

**Affiliations:** 1 Department of Neurosciences and Psychiatry, University of Toledo College of Medicine and Life Sciences, Toledo, OH 43606, USA; 2 Department of Biomedical Informatics and Utah Clinical and Translational Science Institute, University of Utah Spencer Fox Eccles School of Medicine, Salt Lake City, UT 84115, USAjulio.facelli@utah.edu (J.C.F.); davide.jones@utah.edu (D.E.J.); 3 Department of Neurology, University of Utah Spencer Fox Eccles School of Medicine, Salt Lake City, UT 84115, USA; 4 National Center for Advancing Translational Sciences (NCATS), National Institutes of Health (NIH), Rockville, MD 20892, USA

**Keywords:** spinocerebellar ataxia type 2, *ATXN2* gene, high-throughput screening, machine learning, small molecule drug discovery

## Abstract

Spinocerebellar ataxia type 2 (SCA2) is an autosomal dominant neurodegenerative disorder marked by cerebellar dysfunction, ataxic gait, and progressive motor impairments. SCA2 is caused by the pathologic expansion of CAG repeats in the ataxin-2 (*ATXN2*) gene, leading to a toxic gain-of-function mutation of the ataxin-2 protein. Currently, SCA2 therapeutic efforts are expanding beyond symptomatic relief to include disease-modifying approaches such as antisense oligonucleotides (ASOs), high-throughput screening (HTS) for small molecule inhibitors, and gene therapy aimed at reducing *ATXN2* expression. In the present study, data mining and machine learning techniques were employed to analyze HTS data and identify robust molecular properties of potential inhibitors of *ATXN2*. Three HTS datasets were selected for analysis: *ATXN2* gene expression, CMV promoter expression, and biochemical control (luciferase) gene expression. Compounds displaying significant *ATXN2* inhibition with minimal impact on control assays were deciphered based on effectiveness (E) values (*n* = 1321). Molecular descriptors associated with these compounds were calculated using MarvinSketch (*n* = 82). The molecular descriptor data (MD model) was analyzed separately from the experimentally determined screening data (S model) as well as together (MD-S model). Compounds were clustered based on structural similarity independently for the three models using the SimpleKMeans algorithm into the optimal number of clusters (*n* = 26). For each model, the maximum response assay values were analyzed, and E values and total rank values were applied. The S clusters were further subclustered, and the molecular properties of compounds in the top candidate subcluster were compared to those from the bottom candidate subcluster. Six compounds with high *ATXN2* inhibiting potential and 16 molecular descriptors were identified as significantly unique to those compounds (*p* < 0.05). These results are consistent with a quantitative HTS study that identified and validated similar small-molecule compounds, like cardiac glycosides, that reduce endogenous ATXN2 in a dose-dependent manner. Overall, these findings demonstrate that the integration of HTS analysis with data mining and machine learning is a promising approach for discovering chemical properties of candidate drugs for SCA2.

## Simple Summary

In the present study, we aimed to identify molecular features of small-molecule compounds capable of inhibiting the expression of the ataxin-2 (*ATXN2*) gene, which is implicated in Spinocerebellar Ataxia Type 2 (SCA2). Using high-throughput screening (HTS) data from over 1300 compounds and analyzing them with data mining and clustering techniques, we grouped compounds based on structure and biological performance. The top-performing cluster revealed 16 molecular descriptors enriched in *ATXN2* inhibitors, including features also found in validated compound classes like cardiac glycosides. This computational framework may reveal key structural patterns for *ATXN2* inhibition, providing a foundation for future drug development and optimization for SCA2.

## 1. Introduction

Spinocerebellar ataxia type 2 (SCA2) is an autosomal dominant neurodegenerative disorder characterized primarily by cerebellar dysfunction [1]. The clinical manifestations of SCA2 include progressive cerebellar syndrome, ataxic gait, dysmetria, painful muscle contractures, and dysphagia [1,2]. The pathogenesis of SCA2 is caused by the expansion of CAG repeats in the ataxin-2 gene (*ATXN2*), resulting in an abnormal, elongated polyglutamine (polyQ) region within the ataxin-2 protein [3,4,5]. This expanded polyQ tract induces new or toxic gain-of-functions for the ataxin-2 protein that preferentially affects certain neuronal populations [6]. Present therapeutic strategies for SCA2 are predominantly supportive, focusing on physical therapy to alleviate symptoms [7]. Efforts to develop molecular treatments, based on small-molecule pharmaceutical agents, have yet to yield successful results and have only shown efficacy in minimally slowing the progression of neurological symptoms, emphasizing the need for novel therapeutic approaches [8].

A promising therapeutic strategy for SCA2 involves targeting the *ATXN2* gene to suppress its expression [2]. Lowering *ATXN2* mRNA abundance has also been shown to influence ATXN2-interacting proteins, which contribute to RNA metabolism and neurodegeneration [2]. Among these, RNA-binding proteins (RBPs) such as transactive response DNA-binding protein, 43 kilodaltons (TDP-43), and staufen double-stranded RNA-binding protein (STAU1) are implicated in SCA2 due to their roles in stress granule dynamics and autophagy [9,10]. Dysregulation of these processes results in the toxic accumulation of mutant ataxin-2, contributing to neurodegeneration.

In SCA2 mouse models, lowering ATXN2 levels normalizes abnormal neurophysiological phenotypes, while reducing even non-mutant *Atxn2* expression has extended the lifespan of mice overexpressing the TDP-43 protein, demonstrating the potential benefits of targeting *ATXN2* in humans [11]. Beyond ATXN2, STAU1 has emerged as an independent therapeutic target. STAU1 is overexpressed in fibroblast cell lines derived from SCA2 patients as well as in disease models, and its overabundance impairs autophagic flux, further exacerbating protein accumulation and cellular dysfunction. In cell culture models, STAU1 overexpression disrupts autophagy, while STAU1 loss-of-function mouse models show significant decreases in autophagy markers [10].

While antisense oligonucleotides (ASOs) targeting STAU1 have been proposed as a potential mRNA-directed therapy for SCA2, ATXN2-targeting ASOs are already being evaluated in clinical trials [12]. For instance, the Phase 1/2 ALSpire trial (NCT04494256) assessed BIIB105, an investigational ASO designed to reduce ataxin-2 protein levels and mitigate TDP-43 protein aggregation in Amyotrophic Lateral Sclerosis (ALS) [13,14]. Although BIIB105 successfully lowered ataxin-2 levels, the trial did not demonstrate significant clinical benefits, leading to its discontinuation in May 2024.

In addition to ASOs, high-throughput screening (HTS) and gene therapy approaches continue to offer promising avenues for SCA2 therapeutic intervention. HTS is a critical tool in drug discovery [15], enabling the assessment of numerous potential biological modulators and chemical compounds against specific targets without prior knowledge of the compounds’ effects [16]. This method is particularly advantageous in identifying active compounds from extensive libraries, a task that becomes increasingly challenging as the dataset size grows. Given the vast and complex data generated by HTS, in silico data mining and machine learning methods have become indispensable in analyzing this information [17,18]. These computational techniques have proven effective in guiding the design of small pharmaceutical compounds and have shown promise in processing and analyzing HTS data.

Here, we utilized datasets that previously performed HTS and employed unsupervised machine learning (i.e., clustering) and data mining (i.e., feature extraction and descriptor analysis) techniques to identify molecular properties associated with potential small-molecule therapeutic agents for SCA2 [19]. We utilized these HTS datasets to isolate chemical properties among compounds that displayed a high potential for *ATXN2* inhibition. Specifically, we selected three HTS datasets for analysis: expression analysis of the *ATXN2* gene fused with luciferase, expression analysis of the CMV promoter with luciferase, and biochemical control analysis of luciferase expression. For each compound across these datasets, molecular descriptors were evaluated and grouped based on similar structural features using SimpleKMeans clustering. The clusters were subsequently evaluated based on their ability to inhibit *ATXN2* expression while minimizing off-target effects using criteria such as the E value and total rank value.

By utilizing HTS and computational approaches, we identified shared molecular properties of a cluster of small-molecule compounds exhibiting significant potential for inhibiting ATXN2 expression with minimal off-target impacts. Furthermore, these compounds and molecular descriptors are in line with those of validated classes of small-molecule ATXN2-inhibiting compounds identified in a recent study, such as cardiac glycosides [19]. These results corroborate the effectiveness of our computational approach in identifying *ATXN2* inhibitors and establish a framework for further optimization of lead compounds, bringing us closer to developing targeted therapeutics for SCA2.

## 2. Materials and Methods

### 2.1. Pre-Processing and Selection of High-Throughput Screening (HTS) Data for Analysis

The HTS compound screen is described in detail in Scoles et al. [19]. Briefly, HTS datasets (*n* = 7) were obtained from the National Center for Advancing Translational Sciences Chemical Genomics Center (NCGC). These data belong to the assay project titled “qHTS for Inhibitors of ATXN expression” and are available in PubChem under the Bioassay Record AID 588380.

Based on our primary interest in assessing the inhibition of the *ATXN2* gene, three datasets were selected for analysis: expression analysis of the ATXN2 promoter with luciferase (assay 1), expression analysis of the CMV promoter with luciferase (assay 2), and biochemical function assay to determine if compounds inhibited its enzymatic activity (assay 3). The ATXN2 assay quantified expression of the ATXN2 promoter via luciferase-driven luminescence after the cells had been exposed to library compounds. The CMV assay served as a control assay, confirming that compounds were not general luciferase inhibitors. The biochemical control analysis determined whether the luciferase readout across the assays was due to inhibition of luciferase.

Next, the compounds in the three selected assays were filtered. Compounds that were common to all three assays were selected for downstream analysis. A compound that was not present in all three datasets was excluded. A composite dataset was created that included the following: maximum response values and the sample concentration associated with these values for each compound in assay 1, and maximum response values at these concentrations from assay 2 and assay 3 for each compound. The maximum response values were used to calculate the effectiveness (E) value, detailed below. They represent the largest effect of a compound in each assay, indicating its degree of inhibition or activation relative to a baseline.

Compounds in the composite dataset were filtered based on the assignment of an E value to ensure they were specific *ATXN2* inhibitors. The E value was computed by comparing the experimentally determined data with baseline or predicted values to normalize results and ensure that the observed effects were due to the compounds’ biological activity rather than experimental variability. Step-by-step criteria for filtering the compounds are summarized in Table 1. Briefly, the E value assignment was based on two principles: (I) a negative maximum response value in assay 1 ensured that the compound inhibited the *ATXN2* gene, and (II) a negative maximum response value in assay 2 ensured that the compound was a selective inhibitor of mammalian *ATXN2*. If the maximum response value was zero or greater in assay 1, the initial “e” value was set to “0” and the compound was excluded. If the maximum response value was less than 0 in assay 1 but the maximum response value was zero or greater in assay 2, “e” was again set to “0” and the compound was excluded. If the maximum response value was less than zero in assay 1 and in assay 2 (i.e., both principles described above were true), a positive “e” value was assigned by subtracting the absolute values of assay 2 from assay 1. The absolute value of assay 3 was added to this value for a final E value.

For each compound in the filtered dataset, molecular descriptors (i.e., atom count, mass, aliphatic bond count, etc.) were calculated using MarvinSketch version 15.7.27 (ChemAxon, Budapest, Hungary), a chemical drawing and molecular descriptor calculator program [20]. Molecular descriptors were calculated by dividing the “atom type” or “bond type” value by the “total atom” or “total bond” count value, respectively. A list of all possible molecular descriptors is reported in Supplementary Table S1.

### 2.2. Machine Learning Application to the HTS Data and Initial Clustering Analysis

The data for the chemical compounds, including the information calculated for the molecular descriptors and related properties, was uploaded into the Waikato Environment for Knowledge Analysis (WEKA) version 3.7.12 (University of Waikato, Hamilton, New Zealand), an open-source software suite for machine learning and data mining tasks [21]. WEKA was selected due to its well-established use in chemoinformatics, particularly for clustering molecular descriptors in HTS datasets [22,23,24]. Given the structured nature and manageable size of our dataset, WEKA provided an efficient and interpretable framework for analyzing molecular patterns without the need for more computationally intense methods. Three independent analyses were performed on these data: (I) analysis of the calculated molecular descriptor data across all three assays (MD model); (II) analysis of the molecular descriptor and experimentally determined screening data across all three assays (MD-S model); and (III) analysis of the experimentally determined screening data across all three assays (S model). The MD model gave insight into how the chemical structures of the compounds were associated with *ATXN2* inhibition potential (i.e., without considering how the compounds performed in the assays); the S model gave insight on the performance of the compounds as *ATXN2* inhibitors (i.e., which compounds had the strongest biological effect on *ATXN2* inhibition); and the MD-S model gave insight on how the structure and performance of the compounds affected *ATXN2* inhibition.

To determine groups by similarity, the k-means clustering technique (SimpleKMeans algorithm) was applied to the compounds in each model, which minimizes the average squared distance between points in the same cluster [25]. SimpleKMeans was selected for data analysis due to the low complexity of the clusters and the size of the dataset utilized in this study. The number of clusters (*k*) was calculated using the following equation, where N is the total number of samples in the dataset [26]:

(1) $$k=\sqrt{N/2}$$

This well-established formula was selected due to its efficiency in estimating cluster numbers for medium-sized datasets with structured feature spaces, as is typical in HTS datasets [27]. This also provided a computationally simple and effective clustering framework without requiring iterative parameter tuning. To quantitatively evaluate each cluster within a clustering model, a new E value was calculated by averaging the E values from the three assays. Compounds in clusters with a positive E value were candidate clusters with quality compounds (i.e., the greater the E value, the better the inhibiting potential of the compounds within the cluster). Promising clusters that received more positive E values were those with high negative *ATXN2* expression assay values, moderately negative CMV expression assay values, and low negative biochemical control expression assay values. In addition, each cluster was assigned a total rank value. The mean assay values for clusters in the *ATXN2* assay were ranked from the smallest to the largest, whereas the mean values for clusters in the CMV and biochemical assays were ranked from the largest to the smallest. The sum of these three values was the total rank value. A smaller total rank value, negatively correlated with the E value, indicated a greater inhibiting potential of the compounds within the cluster.

### 2.3. Compounds and Associated Molecular Descriptors Within Clusters of Interest

To evaluate the similarity between two clusters from different models (MD, MD-S, and S), a cluster similarity value was calculated for each cluster based on the following formula, where A and B are clusters in different clustering models:

(2) $$cluster\;similarity\;value\;(A,B)=\frac{Number\;of\;compounds\;(A\cap B)}{Number\;of\;compounds\;(A\cup B)}$$

The similarity value is calculated as the number of compounds in both clusters (intersection) divided by the number of total compounds in both clusters (union). This value ranges from 0 to 1, with 0 representing the lowest similarity and 1 representing 100% similarity in terms of the compounds in each clustering model. Cluster similarity values were calculated for two comparisons: one comparing compounds from the MD and MD-S analyses and one comparing compounds from the MD and S analyses. Once cluster similarity values were established, the compounds in the MD and S model clusters were statistically analyzed to establish shared molecular descriptor and screening data similarities.

### 2.4. Subcluster and HTS Data Analysis

Further clustering was performed on the S model clusters to isolate a subset of compounds with shared functional (i.e., *ATXN2* inhibition) and structural properties. To determine the optimal number of subclusters for each S cluster, the SimpleKMeans clustering algorithm was run iteratively, increasing the number of clusters from 1 to 10. For each iteration, the root mean square (RMS) error value was recorded.

A “number of clusters” vs. “RMS error values” graph was generated for each cluster, and the elbow of the graph was analyzed to determine the optimal number of subclusters. Once the appropriate number of subclusters was identified, the SimpleKMeans clustering algorithm was utilized again on the subclusters to further isolate compounds. The RMS error values, which gave insight into the variance within each subcluster and shared structural properties, were recorded and plotted. Subsequently, the standard deviations associated with the mean E values, which gave insight into compounds that had similar functional properties within each subcluster, were plotted. Once plotted, subclusters with low RMS error values and low standard deviations were further analyzed.

### 2.5. Statistical Analysis

**Figure 1:**
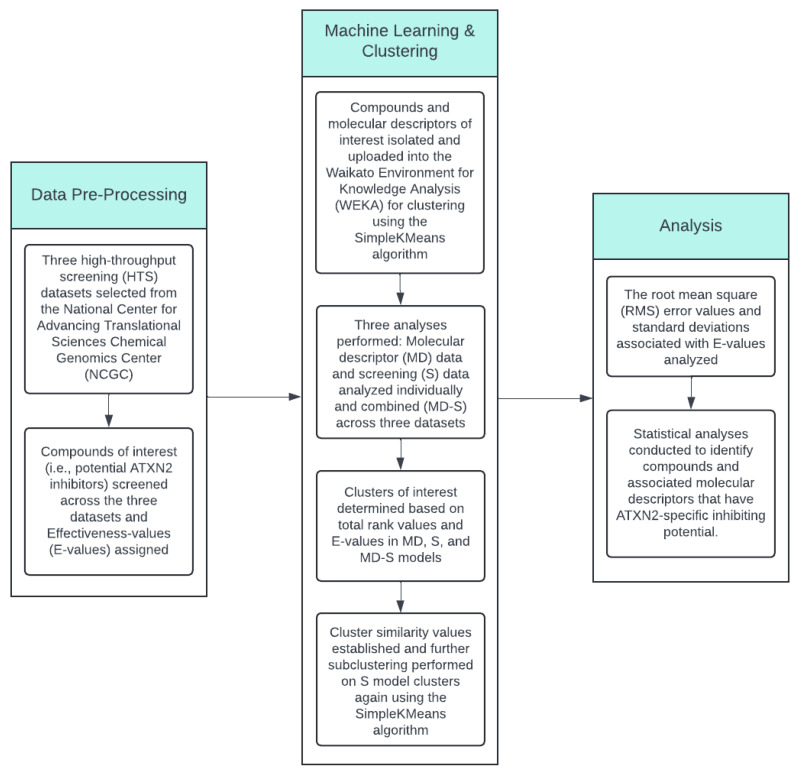
Data analysis workflow. Compounds of interest were pre-processed from the selected high-throughput screening (HTS) datasets. Data mining, machine learning, and statistical analyses were performed to determine the molecular properties associated with potential *ATXN2*-specific inhibitor compounds.

Statistical analyses were conducted to identify molecular descriptors associated with compounds that had *ATXN2*-specific inhibiting potential. Continuous variables (e.g., maximum response assay values, molecular descriptor average values) were presented as mean values ± standard deviations. All data were tested for normality (D’Agostino and Pearson omnibus normality test) and homogeneity of variance (F-test). To determine statistical significance between two variables, data were subjected to a two-tailed unpaired parametric Student’s *t*-test when assumptions for normality and homogeneity of variance were met, a Mann–Whitney non-parametric test when normality was violated, and a Welch’s corrected *t*-test when homogeneity of variance was violated. To account for multiple comparisons and control for the false discovery rate (FDR), a Benjamini–Hochberg correction was applied to *p*-values. The significance level was set at 0.05 for all tests. All statistical analyses were performed using GraphPad Prism version 10.2.3 (GraphPad Software, La Jolla, CA, USA). The comprehensive methods utilized in the present study are concisely diagramed in Figure 1.

## 3. Results

### 3.1. HTS Data for Analysis

Across the three datasets selected for analysis (expression analysis of the *ATXN2* gene with luciferase, assay 1; expression analysis of the CMV promoter with luciferase, assay 2; and biochemical control analysis of the expression of luciferase, assay 3), available in PubChem under Bioassay Record AID 588380, 1407 common compounds were identified. After filtering the data further based on the assignment of E values and the criteria outlined in Table 1, 1321 compounds were predicted to be *ATXN2*-specific inhibitors. After adjusting the ratio of each type of atom or bond in the dataset by dividing the value of each type of atom or bond by its respective total atom or bond count, 82 molecular descriptors emerged for the 1321 compounds using MarvinSuite within MarvinSketch for further analysis.

### 3.2. Data from Machine Learning Application to the HTS Data and Initial Clustering Analysis

After the 1321 compounds and their 82 molecular descriptors were uploaded into WEKA, the SimpleKMeans unsupervised machine learning algorithm was performed. The k-means clustering technique was applied to each model using Equation (1), resulting in *k* = 26 clusters (with an input of *N* = 1321), thereby grouping compounds with similar structural features, in each model: MD, MD-S, and S. For each cluster in each model, the maximum response assay value of the *ATXN2* expression assay and the response values of the CMV and biochemical control expression inhibition at the same concentration of maximum response of the *ATXN2* expression assay were recorded and ranked. The *ATXN2* expression assay values were ranked on a scale of 1 to 26 from smallest to largest. The CMV control expression assay and biochemical control assay values were ranked on a scale of 1 to 26 from largest to smallest. The three rank values were summed for a total rank value, where the lowest total rank value determined the cluster with the best *ATXN2* inhibiting capacity.

The maximum response values ± standard deviations for the three assays, their ranks, number of compounds, E values, and total rank values for each cluster in the MD model are reported in Table 2. The cluster with the smallest number of compounds (*n* = 8) was cluster 10, whereas the cluster with the largest number of compounds (*n* = 98) was cluster 18. The cluster with the largest E value (*E* = 42.74) was cluster 5, followed by cluster 10 (*E* = 21.34). The cluster with the smallest E value was cluster 0 (*E* = −5.16). When assessing the ranks of the maximum response assay values, cluster 5 contained compounds that had the lowest ATXN2 expression assay value (i.e., the greatest *ATXN2* inhibition potential), the highest CMV expression assay value (i.e., the lowest CMV inhibition), and the second highest biochemical expression assay value (i.e., the second lowest luciferase inhibition). Cluster 5 also had the lowest total rank value of 4 among the 26 clusters in the MD model.

The maximum response values ± standard deviations for the three assays, their ranks, number of compounds, E values, and total rank values for each cluster in the MD-S model are displayed in Table 3. The cluster with the smallest number of compounds (*n* = 17) was cluster 14, whereas the cluster with the largest number of compounds (*n* = 115) was cluster 11. The cluster with the largest E value (*E* = 41.66) was cluster 5, followed by cluster 10 (*E* = 18.45). The cluster with the smallest E value was cluster 0 (*E* = −22.96). When assessing the ranks of the maximum response assay values, cluster 0 contained compounds that had the lowest *ATXN2* expression assay value (i.e., the greatest *ATXN2* inhibition), cluster 12 contained compounds that had the highest CMV expression assay value (i.e., the lowest CMV inhibition), and cluster 17 contained compounds that had the highest biochemical expression assay value (i.e., the lowest luciferase inhibition). However, cluster 5 had the lowest total rank value among the 26 clusters in the MD-S model.

The maximum response values ± standard deviations for the three assays, their ranks, number of compounds, E values, and total rank values for each cluster in the S model are displayed in Table 4. The cluster with the smallest number of compounds (*n* = 15) was cluster 0, whereas the cluster with the largest number of compounds (*n* = 127) was cluster 7. The cluster with the largest E value (*E* = 75.06) was cluster 4, followed by cluster 6 (*E* = 50.00). The cluster with the smallest E value was cluster 0 (*E* = −86.80). When assessing the ranks of the maximum response assay values, cluster 21 contained compounds that had the lowest *ATXN2* expression assay value (i.e., the greatest *ATXN2* inhibition), cluster 12 contained compounds that had the highest CMV expression assay value (i.e., the lowest CMV inhibition), and cluster 4 contained compounds that had the highest biochemical expression assay value (i.e., the lowest luciferase inhibition). However, cluster 4 had the lowest total rank value among the 26 clusters in the S model.

### 3.3. Analysis of Compounds and Associated Molecular Descriptors Within Clusters of Interest

Using Equation (2), the cluster similarity value was calculated between the cluster with the lowest total rank value, or highest E value, within each model for cluster pairs. These were clusters 5, 5, and 4 in the MD, MD-S, and S models, respectively (Table 2, Table 3 and Table 4). The cluster similarity value between the compounds within cluster 5 in the MD model and cluster 5 in the MD-S model was 0.89, indicating relatively high similarity. The cluster similarity value between the compounds within cluster 5 in the MD model and cluster 4 in the S model was 0.18, indicating relatively low similarity. The MD and MD-S models contained 16 and 18 compounds, respectively, and all 16 compounds in the MD model were contained within the MD-S model.

Cluster 5 in the MD model was compared to the other 25 MD model clusters. For each assay, the maximum response assay value for cluster 5 vs. the average maximum response assay value for the remaining 25 clusters was analyzed. The *ATXN2* expression assay average maximum response value was significantly different for cluster 5 (−88.56) compared to the average of the remaining clusters (−76.03) (Welch’s corrected t_(21)_ = 5.48, *p* < 0.0001). The CMV expression assay average maximum response value was significantly different for cluster 5 (−43.52) compared to the average of the remaining clusters (−61.02) (Welch’s corrected t_(15)_ = 2.34, *p* = 0.033). The biochemical control expression assay average maximum response value was also significantly different for cluster 5 (−2.30) compared to the average of the remaining clusters (−9.14) (Welch’s corrected t_(20)_ = 2.54, *p* = 0.020).

For all molecular descriptors characterizing the compounds (*n* = 16) in cluster 5 in the MD model, the average value of the molecular descriptors for cluster 5 was compared to the average value of the molecular descriptors for the compounds (*n* = 1305) in the remaining 25 clusters. The molecular descriptors that significantly characterized the 16 cluster 5 compounds were size, polarizability, and hydrophobicity. Cluster 5 contained larger compounds with an average mass of 623.50 Daltons compared to an average mass of 386.10 Daltons for the remaining clusters (Welch’s corrected t_(15)_ = 7.15, *p* < 0.0001), more polarizable compounds with an average molecular polarizability value of 63.78 Å^3^ compared to an average polarizability value of 40.30 Å^3^ for the remaining clusters (Welch’s corrected t_(15)_ = 6.92, *p* < 0.0001), and less hydrophobic compounds with an average partition coefficient (logP) of 1.98 compared to an average logP of 3.58 for the remaining clusters (Mann–Whitney U = 3913, *p* < 0.0001). Visual analysis of these compounds confirmed a core structure of large, fused aliphatic rings with attached carbonyl groups.

The compounds in cluster 5 in the MD model were analyzed further with the compounds in cluster 4 in the S model. From the two clusters, six compounds were present in both clusters, while ten compounds were exclusive to cluster 5 in the MD model. The average maximum response assay value for the overlap compounds (*n* = 6) was calculated and compared to the average maximum response assay value for the 10 MD compounds (*n* = 10) across the three assays. The *ATXN2* expression assay average maximum response value was significantly different between the overlap compounds (−94.36) compared to the MD exclusive compounds (−85.08) (Welch’s corrected t_(9)_ = 3.27, *p* = 0.009). The CMV expression assay average maximum response value was significantly different between the overlap compounds (−12.75) compared to the MD exclusive compounds (−61.98) (Welch’s corrected t_(9)_ = 7.18, *p* < 0.0001). The biochemical control expression assay average maximum response value was significantly different between the overlap compounds (2.99) compared to the average of the remaining clusters (−5.48) (Welch’s corrected t_(10)_ = 2.23, *p* = 0.049).

The molecular descriptors characterizing the overlap compounds were further analyzed by comparing the average molecular descriptor values between the overlap compounds (*n* = 6) and MD exclusive compounds (*n* = 10). The molecular descriptors that significantly characterized the overlap compounds were a greater number of aliphatic rings and a smaller number of aromatic rings. The overlap compounds contained compounds with an average of 6.17 aliphatic rings compared to an average of 3.50 rings in the MD compounds (Mann–Whitney U = 7, *p* = 0.007) as well as an average of 0.17 aromatic rings compared to an average of 1.40 rings in the MD compounds (Welch’s corrected t_(12)_ = 3.03, *p* = 0.010).

### 3.4. Subcluster Analysis

To further decipher molecular descriptors of interest, the S model clusters were subclustered utilizing the SimpleKMeans algorithm. The resulting RMS error values were plotted to determine the optimal number of subclusters based on the “elbow” in each graph (Supplementary Figure S1). These graphs showed an elbow at “2” on the *x*-axis for all clusters, indicating that the optimal number of subclusters for the S model clusters was two.

Once the two subclusters were isolated for each cluster, the SimpleKMeans algorithm was utilized again to determine the RMS error values (Supplementary Figure S2) as well as the standard deviation associated with the E values for each subcluster (Supplementary Figure S3). The first subcluster within cluster 4 (subcluster 4_0), containing six compounds, and the second subcluster within cluster 24 (subcluster 24_1), containing seven compounds, had the desired low RMS error and standard deviation values and were thus analyzed further. Specifically, subcluster 4_0 had an RMS error value of 105.5 and a standard deviation of 2.9. Subcluster 24_1 had an RMS error value of 138.9 and a standard deviation of 3.6. The RMS error values and standard deviations for all subclusters are reported in Supplementary Table S2. Subclusters that had relatively low RMS error values, such as subcluster 0_1 with an RMS of 128.4 or subcluster 11_1 with an RMS of 115.5, were not selected for further analysis since they had relatively higher standard deviations of 34.0 and 18.1, respectively.

Next, the six compounds in subcluster 4_0 and the seven compounds in subcluster 24_1 were visually compared. Based on the molecular descriptors identified in the clustering analysis, subcluster 4_0 contained compounds that exhibited a high potential to inhibit *ATXN2,* whereas cluster 24_1 contained compounds that had low potential to inhibit *ATXN2*. The defining characteristic of the compounds in subcluster 4_0 was a greater number of aliphatic rings, whereas in subcluster 24_1, the defining characteristic was a greater number of aromatic rings.

To understand which molecular descriptors differentiated subcluster 4_0 from 24_1, the means and standard deviations were calculated for each molecular descriptor and compared utilizing appropriate *t*-tests. Analysis of the molecular descriptors associated with the 13 collective compounds from both subclusters revealed 16 molecular descriptors that were significantly different between the two groups. The molecular descriptors that significantly characterized and differentiated subcluster 4_0 were: high aliphatic ring count, asymmetric atom count, carbo ring count, chiral center count, aliphatic ring ratio, asymmetric atom ratio, carboaliphatic ring ratio, chiral center ratio, low aromatic atom count, aromatic bond count, aromatic ring count, carboaromatic ring count, aromatic atom ratio, aromatic bond ratio, aromatic ring ratio, and carboaromatic ring ratio (*p* < 0.05 for all, Table 5).

**Figure 2:**
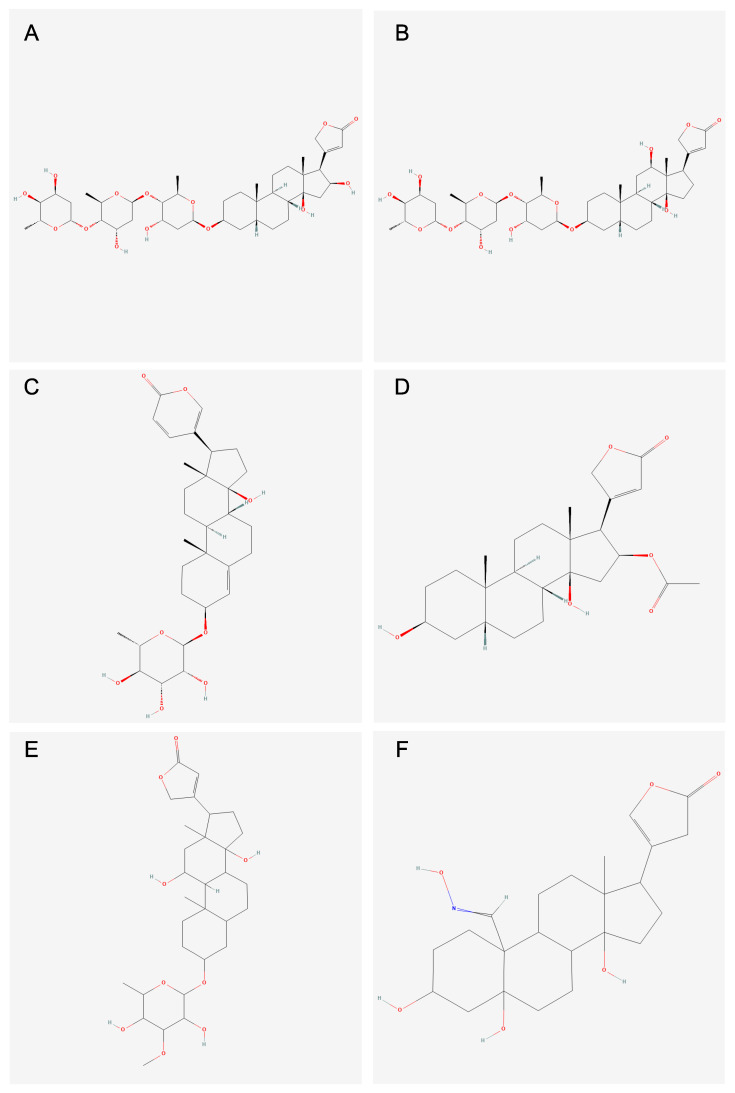
Chemical structures of the six strong potential *ATXN2* inhibitor compounds from subcluster 4_0. Compounds include the five cardiac glycosides (**A**) Gitoxin (C_41_H_64_O_14_), (**B**) Digoxin (C_41_H_64_O_14_), (**C**) Proscillaridin (C_30_H_42_O_8_), (**D**) Oleandrigenin (C_25_H_36_O_6_), and (**E**) Sarnovide (C_30_H_46_O_9_), as well as a steroid derivative of the cardiac glycoside, strophanthidin, (**F**) Strophanthidin oxime (C_23_H_33_NO_6_). These compounds are subclustered based on their filtered E value thresholds and favorable molecular descriptor profiles (e.g., high aliphatic ring count, low aromatic ring count). Their cumulative performance across these parameters supports their prioritization for further experimental validation.

Cluster 24_1 contained compounds with high aromatic ring count, aromatic atom count, aromatic bond count, and carboaromatic ring ratio. Cluster 4_0 contained the following six compounds: Gitoxin (PubChem CID: 91540), Digoxin (PubChem CID: 2724385), Proscillaridin (PubChem CID: 5284613), Oleandrigenin (PubChem CID: 9802865), Sarnovide (PubChem CID: 276294), and Strophanthidin oxime (PubChem CID: 4024368). These compounds shared molecular descriptors such as high aliphatic ring count, asymmetric atom count, carbo ring count, and chiral center count. The chemical structures of the six compounds in cluster 4_0 are shown in Figure 2. The chemical structures of the seven compounds in cluster 24_1 are reported in Supplementary Figure S4.

## 4. Discussion

The results of this study build upon the existing body of research on SCA2 by utilizing HTS data to identify molecular descriptors associated with *ATXN2* inhibitor compounds. Studies spanning the last 30 years have extensively characterized the pathological mechanisms of SCA2, particularly the toxic gain-of-function caused by the CAG repeat expansion in the *ATXN2* gene [28,29,30,31]. This study, utilizing HTS and computational analysis integration, contributes to the therapeutic research for SCA2 by focusing on the molecular profiling of small-molecule candidate compounds that exhibit high *ATXN2* inhibition potential, complementing approaches reducing ATXN2 abundance with ASOs.

A key aspect of this work was the clustering of compounds based on their *ATXN2* inhibition, CMV control, and biochemical control assay values. By utilizing the SimpleKMeans clustering algorithm within WEKA [32], our analysis successfully clustered compounds isolated from the three assays into 26 clusters for three analysis models (Table 2, Table 3 and Table 4). The analysis of molecular descriptor data (MD model) and screening data (S model) independently and combined (MD-S model) enabled us to prioritize compounds predicted to be selective *ATXN2* inhibitors.

The low cluster similarity value of 0.18 between the candidate clusters in the MD (cluster 5) and S (cluster 4) models indicated low similarity between the chemical structures and performance of ATXN2 inhibitor compounds; thus, these clusters were compared to scrutinize their molecular properties. The compounds in cluster 5 in the MD model were differentiated from the other MD clusters by their larger size, greater polarizability, and lower hydrophobicity, which align with established general principles in drug discovery that indicate larger and more polarizable molecules engage in stronger interactions with biological targets [18].

The compounds that overlapped in cluster 5 from the MD model and cluster 4 in the S model were characterized by a greater number of aliphatic rings and a smaller number of aromatic rings. This finding implies that flexibility in molecular structure may be a key factor in *ATXN2* inhibition since aliphatic rings tend to be more flexible and less rigid with their open chain structures compared to the stable, planar ring structures characterized by alternating double and single bonds (i.e., benzene) in aromatic rings [33,34,35]. This flexibility may enable better adaptation and interaction with the ataxin-2 protein, enhancing the inhibitory effect [33]. In addition, the reduction in aromatic rings may decrease non-specific interactions, possibly reducing off-target effects and improving selectivity [36].

To more precisely understand the molecular properties driving the biological effect of *ATXN2* inhibition, the 26 S model clusters were further subclustered. Visual comparison of the candidate subclusters, based on RMS error and standard deviation of E values, revealed that subcluster 4_0 contained compounds with a high number of aliphatic rings (Figure 2). Furthermore, this subcluster had the highest *ATXN2*-inhibition potential relative to all compounds in the analysis and was differentiated by 16 molecular descriptors (Table 5). The top features of these compounds were high asymmetric atom counts, carbo ring counts, chiral center counts, chiral center ratio, aliphatic ring ratio, and carboaliphatic ring ratio, in addition to the high aliphatic ring counts previously established.

Notably, five of the six compounds in subcluster 4_0 included the Na^+^/K^+^-ATPase-inhibiting cardiac glycosides proscillaridin, gitoxin, digoxin, oleandrigenin, and sarnovide. These compounds are characterized by a steroid-like core structure with multiple sugar (glycoside) attachments. Their defining molecular features include high aliphatic ring count, carboaliphatic ring count, chiral center count, asymmetric atom count, carbo ring count, aliphatic ring count, and chiral center ratio [20,37,38,39,40,41]. The sixth compound, although not a classic cardiac glycoside, was strophanthidin oxime, which is a steroid derivative of the cardiac glycoside strophanthidin comprising similar molecular features [42].

These properties suggest that the compounds that are highly tailored for *ATXN2* inhibition are characterized by several key chemical features that enhance their biological activity. These features include their stereochemistry and specific ring systems in addition to their structural flexibility. The presence of asymmetric atoms (i.e., chiral centers) further contributes to their specificity, as these atoms may produce stereoisomers with different spatial configurations, enhancing binding efficacy [43]. In addition, the high carboaliphatic and carbo ring counts reinforce the non-aromatic, flexible nature of these molecules [44], perhaps further aiding in their adaptability to the ataxin-2 protein. The compounds’ elevated chiral center count suggests increased potential for selective interactions, reducing off-target effects [45]. Lastly, the fused aliphatic rings offer a balance between rigidity and flexibility, contributing to the compounds’ ability to maintain structural integrity while fitting into the binding site [46], all of which together may contribute to their potential as strong *ATXN2* inhibitors.

The significant difference in molecular descriptors between high- and low-performing clusters emphasizes the importance of structural features in the efficacy of potential *ATXN2* inhibitors. In addition, the integration of HTS with data mining and machine learning allowed for a high-throughput evaluation of thousands of compounds, similar to methods described by Inglese et al. [15] in earlier screening efforts. The large-scale nature of this study adds robustness to our findings, further validating the identified molecular descriptors as key factors in determining *ATXN2* inhibition potential.

Importantly, the candidate compounds identified through our methods correspond with in vitro-confirmed small-molecule ATXN2 inhibitors reported in a recent study by Scoles et al. [19]. This study screened over 400K compounds, which included the subset utilized in the present study, using a multiplexed luciferase reporter assay in human embryonic kidney 293 (HEK-293) cells, leading to the identification of two major compound classes capable of lowering ATXN2 levels: HSP90 inhibitors and cardiac glycosides. Treatment of mice expressing the human ATXN2 gene with an HSP90 inhibitor, 17-demethoxygeldanamycin (17-DMAG), resulted in a 90% reduction in ATXN2 protein levels in the cerebellum, accompanied by improvements in autophagy markers like STAU1. Similarly, proscillaridin A, a cardiac glycoside, reduced *ATXN2* transcription in a dose-dependent manner in HEK-293 cells.

HSP90 inhibitors typically contain highly conjugated structures with aromatic rings and often target adenosine triphosphate (ATP) binding sites through planar aromatic scaffolds. Although these compounds were not identified in our top-performing subcluster, their defining molecular features include high aromatic ring count, aromatic atom count, aromatic bond count, carboaromatic ring count, aromatic ring ratio, aromatic atom ratio, and aromatic bond ratio, which partially align with the top chemical features identified in our analysis [20,47].

In summary, the study offers a novel computational framework for identifying molecular properties of *ATXN2* inhibitors. Future work will involve applying our methodology, or more advanced machine learning methods, to perform further computational chemistry structure-to-function studies on the larger quantitative HTS dataset utilized in the bench validation study described [19] as well as in other publicly available HTS datasets in PubChem [48]. Future work should also continue to experimentally validate the top-performing compounds identified by computational methods to explore the development of structure-activity relationships and refine these compounds further. This research lays the groundwork for the discovery of disease-modifying treatments for SCA2, a significant unmet need in current therapeutic strategies [49].

## 5. Limitations

While this study presents a novel approach for molecularly characterizing *ATXN2* inhibitors using HTS and machine learning, some limitations should be considered.

The effectiveness (E) values calculated for each compound are based on data from three assays (*ATXN2* expression, CMV control, and biochemical control). While this design aims to control for off-target effects, the assays themselves may exhibit variability due to factors like assay sensitivity, compound stability, or experimental conditions [50], which may affect the reproducibility of the results and needs to be controlled in experimental validation studies.

The study’s focus on molecular descriptors related to compound structure may overlook important functional dynamics such as protein–ligand interactions. While structural clustering provides insight into potential *ATXN2* inhibitors, additional computational approaches, like molecular docking [51] or dynamic simulations [52], may more holistically capture the binding affinities and interactions at the molecular level.

The reliance on computational methods for clustering and analyzing compounds may introduce biases inherent in algorithmic predictions. Although the SimpleKMeans algorithm effectively grouped compounds based on molecular descriptors, its performance is sensitive to initial conditions and may not capture all potential interactions between compounds and biological targets [53]. This limitation highlights the importance of continuing to validate these findings through in vitro and in vivo assays to confirm the predicted *ATXN2* inhibition activity.

Finally, the inhibitory potential of compounds identified in this study is based solely on their performance in an HTS setting; however, translating these findings into clinically effective treatments remains challenging, as many compounds that demonstrate efficacy in screening assays fail to produce similar results when used in secondary in vitro screens, preclinical animal models, as well as in clinical trials due to bioavailability, toxicity, or off-target effects [54]. A future step will involve conducting pharmacokinetic and pharmacodynamic assessments to evaluate the suitability of these compounds for human trials.

## 6. Conclusions

This study successfully demonstrated the utility of integrating HTS, data mining, and machine learning to identify molecular descriptors linked to potent *ATXN2* inhibition. By clustering compounds based on their structural features and *ATXN2* inhibition capacity, we identified six compounds and 16 molecular descriptors that are associated with high inhibition potential. These findings not only contribute to the understanding of structure-based drug discovery for SCA2 but also provide a promising foundation for continued experimental validation. Future work should focus on advancing the computationally identified compounds into clinical trials to assess their therapeutic potential in humans.

## Figures and Tables

**Table 1:** Criteria applied to assign effectiveness (E) values to compounds of interest.

1. If (ATXN ≥ 0)	e = 0
2. If (ATXN < 0)	
and if (CMV ≥ 0)	e = 0
3. If (ATXN < 0)	
and if (CMV < 0)	e = |ATXN| − |CMV|
4. If (e = 0)	E = 0
5. If (e > 0)	
and if (Luc < 0)	E = e − |Luc|
6. If (e > 0)	
and if (Luc ≥ 0)	E = e + |Luc|

Abbreviations: ATXN = ataxin-2 gene, CMV = cytomegalovirus control, Luc = biochemical control.

**Table 2:** Maximum response assay values and ranks for the *ATXN2* expression assay, CMV control assay, and biochemical control expression assay, as well as the number of compounds, E values, and total rank values for the 26 clusters from the molecular descriptor (MD) data analysis.

Cluster	ATXN2 Expression Assay	Rank 1	CMV Control Assay	Rank 2	Biochemical Control Assay	Rank 3	Number of Compounds	E	Total Rank
**0**	−70.95 ± 28.08	23	−55.33 ± 32.05	6	−20.78 ± 25.76	26	40	−5.16	55
**1**	−73.20 ± 17.10	19	−63.87 ± 18.86	21	−6.71 ± 12.11	12	55	2.62	52
**2**	−74.91 ± 20.58	15	−61.79 ± 20.82	13	−10.23 ± 16.78	16	79	2.89	44
**3**	−68.91 ± 21.68	26	−59.49 ± 22.02	10	−6.46 ± 15.74	9	66	2.96	45
**4**	−80.60 ± 12.49	6	−62.06 ± 15.69	14	−11.75 ± 11.65	20	41	6.79	40
**5**	−88.56 ± 8.32	1	−43.52 ± 29.75	1	−2.30 ± 10.00	2	16	42.74	4
**6**	−80.17 ± 21.66	8	−67.70 ± 25.45	25	−14.04 ± 16.10	23	45	−1.57	56
**7**	−76.12 ± 17.96	12	−55.06 ± 27.56	5	−5.22 ± 16.44	7	54	15.85	24
**8**	−83.22 ± 10.94	3	−65.21 ± 15.89	22	−3.18 ± 14.16	4	24	14.83	29
**9**	−73.65 ± 23.88	17	−51.92 ± 24.17	2	−13.56 ± 20.00	22	70	8.17	41
**10**	−87.49 ± 5.94	2	−66.02 ± 22.92	24	−0.13 ± 3.23	1	8	21.34	27
**11**	−80.73 ± 15.56	5	−63.36 ± 18.92	20	−15.47 ± 16.58	24	92	1.89	49
**12**	−75.96 ± 24.82	13	−62.54 ± 28.54	16	−18.31 ± 21.93	25	52	−4.88	54
**13**	−71.68 ± 18.85	21	−61.17 ± 19.09	12	−6.59 ± 13.02	11	67	3.92	44
**14**	−73.37 ± 20.13	18	−58.32 ± 18.60	9	−11.51 ± 17.49	19	58	3.53	46
**15**	−71.15 ± 24.88	22	−56.03 ± 22.68	7	−12.81 ± 18.74	21	76	2.31	50
**16**	−78.14 ± 19.35	10	−65.68 ± 23.21	23	−9.67 ± 15.88	15	67	2.79	48
**17**	−79.95 ± 21.80	9	−62.77 ± 29.69	17	−3.53 ± 9.14	5	17	13.66	31
**18**	−74.63 ± 18.67	16	−57.77 ± 19.27	8	−11.35 ± 18.14	17	98	5.51	41
**19**	−70.79 ± 18.05	24	−62.89 ± 17.83	19	−6.51 ± 13.14	10	53	1.39	53
**20**	−69.21 ± 22.92	25	−54.83 ± 24.57	4	−3.56 ± 8.39	6	23	10.82	35
**21**	−75.62 ± 23.16	14	−62.52 ± 24.31	15	−8.92 ± 10.92	14	27	4.18	43
**22**	−72.00 ± 20.00	20	−54.04 ± 23.87	3	−3.03 ± 6.77	3	70	14.93	26
**23**	−80.19 ± 13.71	7	−62.82 ± 13.26	18	−11.37 ± 13.88	18	38	6.00	43
**24**	−76.41 ± 24.14	11	−61.05 ± 23.66	11	−8.04 ± 12.60	13	49	7.32	35
**25**	−81.66 ± 14.10	4	−71.27 ± 19.48	26	−5.79 ± 13.92	8	36	4.60	38

The maximum response mean values and standard deviations are reported for each cluster (0 through 25) for each of the assays (*ATXN2* expression assay, CMV control assay, and biochemical control assay), where standard deviations were based on the maximum response value for each compound. The “Rank 1” column indicates the rank of the *ATXN2* expression assay expression from the smallest to the largest. The “Rank 2” column indicates the rank of the CMV control expression assay from the largest to the smallest. The “Rank 3” column indicates the rank of the biochemical control expression assay from the largest to the smallest. The number of compounds in each cluster and E values for each cluster are also reported. The “Total rank” column indicates the sum of Rank 1, Rank 2, and Rank 3. Highlighted row represents the cluster with the highest E value and lowest total rank value, containing compounds of interest for downstream analysis.

**Table 3:** Maximum response assay values and ranks for the *ATXN2* expression assay, CMV control assay, and biochemical control expression assay, as well as the number of compounds, E values, and total rank values for the 26 clusters from the molecular descriptor and experimentally determined screening data analysis (MD-S).

Cluster	ATXN2 Expression Assay	Rank 1	CMV Control Assay	Rank 2	Biochemical Control Assay	Rank 3	Number of Compounds	E	Total Rank
**0**	−95.30 ± 7.22	1	−91.73 ± 9.19	26	−26.53 ± 31.41	25	50	−22.96	52
**1**	−70.27 ± 14.38	18	−53.95 ± 17.21	9	−4.74 ± 8.99	8	48	11.58	35
**2**	−81.17 ± 11.20	12	−62.26 ± 15.96	14	−8.42 ± 14.50	15	77	10.49	41
**3**	−63.42 ± 17.29	23	−53.86 ± 12.23	8	−7.62 ± 10.81	14	74	1.94	45
**4**	−64.23 ± 18.26	22	−28.10 ± 14.27	3	−2.92 ± 9.71	3	56	33.21	28
**5**	−87.94 ± 9.63	3	−43.63 ± 29.00	6	−2.65 ± 9.68	2	18	41.66	11
**6**	−68.12 ± 20.87	21	−40.64 ± 17.02	5	−13.03 ± 16.69	20	35	14.44	46
**7**	−85.69 ± 12.36	6	−71.50 ± 19.28	20	−5.31 ± 17.96	10	43	8.88	36
**8**	−82.96 ± 9.64	10	−66.85 ± 11.52	18	−5.27 ± 12.71	9	55	10.84	37
**9**	−78.08 ± 12.77	15	−56.08 ± 16.81	10	−16.18 ± 19.49	24	68	5.81	49
**10**	−83.05 ± 8.27	9	−60.48 ± 16.58	12	−4.12 ± 9.12	5	18	18.45	26
**11**	−86.72 ± 8.04	4	−71.86 ± 15.09	22	−14.00 ± 15.95	21	115	0.86	47
**12**	−15.33 ± 13.66	26	−12.87 ± 10.45	1	−3.08 ± 8.84	4	22	−0.62	31
**13**	−72.96 ± 15.81	17	−63.07 ± 15.48	16	−4.17 ± 7.63	6	69	5.72	39
**14**	−69.10 ± 17.06	20	−48.02 ± 16.85	7	−42.99 ± 28.42	26	17	−21.91	53
**15**	−29.47 ± 14.34	25	−25.71 ± 16.14	2	−5.97 ± 8.25	11	53	−2.21	38
**16**	−84.98 ± 10.92	7	−71.52 ± 15.56	21	−16.00 ± 21.24	23	59	−2.55	51
**17**	−94.83 ± 6.92	2	−89.92 ± 10.17	25	−1.87 ± 4.93	1	39	3.04	28
**18**	−78.27 ± 13.21	14	−62.09 ± 16.59	13	−11.47 ± 15.57	17	66	4.70	44
**19**	−81.81 ± 11.80	11	−72.52 ± 13.38	23	−7.10 ± 10.45	13	61	2.19	47
**20**	−53.81 ± 19.74	24	−29.08 ± 13.79	4	−11.54 ± 12.92	18	37	13.18	46
**21**	−86.69 ± 13.26	5	−75.62 ± 15.68	24	−10.03 ± 15.17	16	27	1.04	45
**22**	−83.43 ± 12.34	8	−68.40 ± 13.84	19	−14.18 ± 17.41	22	65	0.85	49
**23**	−78.62 ± 10.66	13	−62.66 ± 10.96	15	−12.11 ± 13.55	19	53	3.85	47
**24**	−70.25 ± 15.49	19	−59.12 ± 16.38	11	−4.27 ± 10.47	7	65	6.86	37
**25**	−77.31 ± 16.90	16	−65.69 ± 19.06	17	−6.45 ± 14.83	12	31	5.16	45

The maximum response mean values and standard deviations are reported for each cluster (0 through 25) for each of the assays (*ATXN2* expression assay, CMV control assay, and biochemical control assay), where standard deviations were based on the maximum response value for each compound. The “Rank 1” column indicates the rank of the *ATXN2* expression assay expression from the smallest to the largest. The “Rank 2” column indicates the rank of the CMV control expression assay from the largest to the smallest. The “Rank 3” column indicates the rank of the biochemical control expression assay from the largest to the smallest. The number of compounds in each cluster and E values for each cluster are also reported. The “Total rank” column indicates the sum of Rank 1, Rank 2, and Rank 3. Highlighted row represents the cluster with the highest E value and lowest total rank value, containing compounds of interest for downstream analysis.

**Table 4:** Maximum response assay values and ranks for the *ATXN2* expression assay, CMV control assay, and biochemical control expression assay, as well as the number of compounds, E values, and total rank values for the 26 clusters from the experimentally determined screening data analysis (S).

Cluster	ATXN2 Expression Assay	Rank 1	CMV Control	Rank 2	Biochemical Control	Rank 3	Number of Compounds	E	Total Rank
**0**	−94.39 ± 6.96	4	−93.02 ± 7.79	25	−88.17 ± 10.78	26	15	−86.80	55
**1**	−30.70 ± 7.61	25	−39.44 ± 7.62	7	−5.76 ± 9.41	17	43	−14.51	49
**2**	−70.55 ± 5.02	17	−53.78 ± 4.71	11	−4.34 ± 5.61	13	69	12.43	41
**3**	−47.15 ± 6.30	23	−55.00 ± 4.67	13	−4.69 ± 5.91	14	44	−12.53	50
**4**	−88.57 ± 6.81	5	−12.84 ± 5.72	2	−0.68 ± 7.39	1	24	75.06	8
**5**	−85.39 ± 3.80	9	−73.68 ± 4.22	21	−19.17 ± 5.18	19	74	−7.45	49
**6**	−86.65 ± 6.21	7	−34.77 ± 4.20	6	−1.88 ± 4.03	4	34	50.00	17
**7**	−86.73 ± 3.40	6	−71.17 ± 2.43	20	−2.74 ± 4.06	7	127	12.82	33
**8**	−83.19 ± 5.32	12	−65.60 ± 6.04	17	−38.68 ± 7.78	24	32	−21.09	53
**9**	−69.34 ± 5.10	18	−26.73 ± 7.09	5	−1.77 ± 5.11	3	38	40.84	26
**10**	−79.04 ± 7.55	14	−41.79 ± 6.61	9	−30.33 ± 9.41	20	33	6.93	43
**11**	−95.41 ± 5.41	2	−90.50 ± 7.49	24	−36.86 ± 9.23	23	34	−31.96	49
**12**	−13.14 ± 7.97	26	−12.08 ± 8.07	1	−5.66 ± 8.79	16	38	−4.60	43
**13**	−57.09 ± 4.95	21	−66.84 ± 4.14	18	−3.91 ± 6.36	11	52	−13.67	50
**14**	−70.98 ± 9.53	16	−53.81 ± 10.13	12	−68.46 ± 12.65	25	19	−51.29	53
**15**	−41.83 ± 7.31	24	−16.49 ± 8.09	4	−2.39 ± 7.13	6	36	22.96	34
**16**	−71.70 ± 4.26	15	−70.37 ± 4.81	19	−1.50 ± 5.02	2	87	−0.17	36
**17**	−95.03 ± 3.37	3	−83.68 ± 4.52	23	−3.72 ± 5.76	10	46	7.63	36
**18**	−82.70 ± 4.41	13	−59.62 ± 4.39	15	−18.64 ± 5.42	18	53	4.43	46
**19**	−85.75 ± 4.75	8	−49.89 ± 3.94	10	−3.92 ± 4.88	12	67	31.95	30
**20**	−67.87 ± 12.39	19	−15.96 ± 7.71	3	−32.55 ± 15.15	22	15	19.35	44
**21**	−98.83 ± 2.18	1	−96.97 ± 2.68	26	−3.21 ± 6.01	9	96	−1.35	36
**22**	−62.51 ± 8.13	20	−57.90 ± 6.65	14	−31.95 ± 8.35	21	23	−27.34	55
**23**	−55.05 ± 5.93	22	−40.79 ± 5.93	8	−5.52 ± 8.90	15	54	8.75	45
**24**	−85.38 ± 3.58	10	−79.59 ± 3.18	22	−2.95 ± 5.10	8	68	2.84	40
**25**	−85.14 ± 4.71	11	−61.87 ± −61.87	16	−2.16 ± 6.54	5	100	21.10	32

The maximum response mean values and standard deviations are reported for each cluster (0 through 25) for each of the assays (*ATXN2* expression assay, CMV control assay, and biochemical control assay), where standard deviations were based on the maximum response value for each compound. The “Rank 1” column indicates the rank of the *ATXN2* expression assay expression from the smallest to the largest. The “Rank 2” column indicates the rank of the CMV control expression assay from the largest to the smallest. The “Rank 3” column indicates the rank of the biochemical control expression assay from the largest to the smallest. The number of compounds in each cluster and E values for each cluster are also reported. The “Total rank” column indicates the sum of Rank 1, Rank 2, and Rank 3. Highlighted row represents the cluster with the highest E value and lowest total rank value, containing compounds of interest for downstream analysis.

**Table 5:** Molecular descriptors differentiating subclusters 4_0 and 24_1.

	Molecular Descriptor	Subcluster 4_0 Mean ± SD	Subcluster 24_1 Mean ± SD	Statistical Test Result
**1**	Aliphatic ring count	6.17 ± 1.47	3.57 ± 1.90	Mann–Whitney U = 5.50, *p* = 0.023
**2**	Aromatic atom count	1.00 ± 2.45	10.43 ± 7.83	Welch’s corrected t_(7)_ = 3.02, *p* = 0.018
**3**	Aromatic bond count	1.00 ± 2.45	10.71 ± 7.93	Welch’s corrected t_(7)_ = 3.07, *p* = 0.017
**4**	Aromatic ring count	0.17 ± 0.41	1.86 ± 1.35	Welch’s corrected t_(7)_ = 3.16, *p* = 0.015
**5**	Asymmetric atom count	14.17 ± 5.71	6.00 ± 3.74	Student’s t_(11)_ = 3.100, *p* = 0.010
**6**	Carboaromatic ring count	0.00 ± 0.00	1.43 ± 1.27	Welch’s corrected t_(6)_ = 2.97, *p* = 0.025
**7**	Carbo ring count	4.00 ± 0.00	2.57 ± 1.40	Welch’s corrected t_(6)_ = 2.71, *p* = 0.035
**8**	Chiral center count	14.17 ± 5.71	6.57 ± 3.51	Student’s t_(7)_ = 2.94, *p* = 0.013
**9**	Aliphatic ring ratio	6.17 ± 1.47	3.57 ± 1.90	Welch’s corrected t_(7)_ = 2.93, *p* = 0.022
**10**	Aromatic atom ratio	0.01 ± 0.03	0.11 ± 0.08	Welch’s corrected t_(8)_ = 2.95, *p* = 0.019
**11**	Aromatic bond ratio	0.01 ± 0.03	0.11 ± 0.08	Welch’s corrected t_(8)_ = 3.00, *p* = 0.018
**12**	Aromatic ring ratio	0.03 ± 0.07	0.32 ± 0.26	Welch’s corrected t_(7)_ = 2.93, *p* = 0.022
**13**	Asymmetric atom ratio	0.12 ± 0.02	0.07 ± 0.06	Student’s t_(11)_ = 3.419, *p* = 0.006
**14**	Carboaliphatic ring ratio	0.66 ± 0.13	0.22 ± 0.28	Mann–Whitney U = 3.00, *p* = 0.008
**15**	Carboaromatic ring ratio	0.00 ± 0.00	0.27 ± 0.27	Welch’s corrected t_(6)_ = 2.64, *p* = 0.038
**16**	Chiral center ratio	0.16 ± 0.02	0.08 ± 0.05	Student’s t_(11)_ = 3.35, *p* = 0.007

Significantly different molecular descriptors between subclusters 4_0 and 24_1 (*n* = 16). Molecular descriptor values for subcluster 4_0 and 24_1 are presented as mean ± standard deviation (SD) in columns two and three, respectively. The appropriate statistical test was applied based on whether data met assumptions for normal distribution and homogeneity of variance. Statistical test values and *p*-values are listed in column four, indicating statistical significance (*p* < 0.05). To account for multiple comparisons and control for the false discovery rate (FDR), the Benjamini–Hochberg correction was applied to *p*-values.

## Data Availability

Data are contained within the article and Supplementary Materials. All screening data are available in PubChem (AID number 588380).

## Supplementary Materials

The following supporting information can be downloaded at: https://www.mdpi.com/article/10.3390/biology14050522/s1. Supplementary Table S1, All possible molecular descriptors in MarvinSketch; Supplementary Figure S1, Plots showing the average root mean square (RMS) error values from the SimpleKMeans clustering algorithm for each cluster in the S model; Supplementary Figure S2, root mean square (RMS) error values for all subclusters within each screening (S) model cluster from the SimpleKMeans algorithm; Supplementary Figure S3, Standard deviations associated with E values for all subclusters within each screening (S) model cluster; Supplementary Table S2, root mean square (RMS) error values as well as mean E values and standard deviations associated with mean E values for each subcluster from the screening only (S) clusters; Supplementary Figure S4, Chemical structures of the seven weak ATXN2 inhibiting compounds from subcluster 24_1.
