# Supplementary material for: Identifying Molecular Properties of Ataxin-2 Inhibitors for Spinocerebellar Ataxia Type 2 Utilizing High-Throughput Screening and Machine Learning
Source: Biology (Basel). 2025 May 8;14(5):522. doi: 10.3390/biology14050522 (PMC12108740; doi:10.3390/biology14050522)
Supplement: Supplementary file 1 [file biology-14-00522-s001.zip › biology-3627433-supplementary.pdf]

Supplementary Materials | Identifying Molecular Properties of Ataxin-2 Inhibitors for Spinocerebellar Ataxia Type 2 utilizing High-Throughput Screening and Machine Learning

Contents:

Supplementary Table 1	Page 2
Supplementary Figure 1	Page 4
Supplementary Figure 2	Page 13
Supplementary Figure 3	Page 14
Supplementary Table 2	Page 15
Supplementary Figure 4	Page 17

Supplementary Table 1. All possible molecular descriptors in MarvinSketch.

**Atom & Bond Descriptors**	
**Aliphatic atom count**	Number of atoms in the molecule having no aromatic bond (excluding hydrogens)
**Aliphatic bond count**	Number of non-aromatic bonds in the molecule (excluding bonds of hydrogen atoms)
**Aromatic atom count**	Number of atoms in the molecule having aromatic bonds
**Aromatic bond count**	Number of aromatic bonds in the molecule
**Asymmetric atom count**	Number of asymmetric atoms (having four different ligands)
**Atom count**	Number of atoms in the molecule including hydrogens
**Bond count**	Number of bonds in the molecule including bonds of hydrogen atoms
**Chain atom count**	Number of chain atoms (non-ring atoms excluding hydrogens)
**Chain bond count**	Number of chain bonds (non-ring bonds excluding bonds of hydrogen atoms)
**Chiral center count**	Number of tetrahedral stereogenic centers. This function identifies two chiral centers in 1,4-dimethylcyclohexane, which does not contain asymmetric atoms
**Ring atom count**	Number of ring atoms
**Ring bond count**	Number of ring bonds
**Rotatable bond count**	Number of rotatable bonds in the molecule. Unsaturated bonds, and single bonds connected to hydrogens or terminal atoms, single bonds of amides, sulphonamides and those connecting two hindered aromatic rings (having at least three ortho substituents) are considered non-rotatable
**Stereo double bond count**	Number of double bonds with defined stereochemistry
**Ring Descriptors**	
**Aliphatic ring count**	Number of those rings in the molecule that have non-aromatic bonds (SSSR based)
**Aromatic ring count**	Number of aromatic rings in the molecule. This number is calculated from the smallest set of smallest aromatic rings (SSSAR), which might contain rings which are not part of the standard SSSR ring set. Therefore, the sum of the aliphatic ring count and the aromatic ring count can sometimes be greater than the ring count value. The difference is the signal of a macroaromatic ring system
**Carbo ring count**	Number of rings containing only carbon atoms
**Carboaliphatic ring count**	Number of aliphatic rings containing only carbon atoms
**Carboaromatic ring count**	Number of aromatic rings containing only carbon atoms (SSSAR based)

**Fused aliphatic ring count**	Number of aliphatic rings having common bonds with other rings
**Fused aromatic ring count**	Number of aromatic rings having common bonds with other rings
**Fused ring count:**	Number of fused rings in the molecule (having common bonds)
**Hetero ring count**	Number of rings containing hetero atom(s)
**Heteroaromatic ring count**	Number of aromatic heterocycles in the molecule
**Largest ring size**	Size of the largest ring in the molecule
**Largest ring system size**	Number of rings in the largest ring system
**Ring count**	Number of rings in the molecule. This calculation is based on SSSR (Smallest Set of Smallest Rings)
**Ring system count**	Number of disjunct ring systems
**Smallest ring size**	Size of the smallest ring in the molecule
**Smallest ring system size**	Number of rings in the smallest ring system
**Path-Based Descriptors**	
**Platt index**	Sum of the edge degrees of a molecular graph
**Randic index**	Harmonic sum of the geometric means of the node degrees for each edge
**Distance-Based Descriptors**	
**Balaban index**	The Balaban distance connectivity of the molecule, which is the average distance sum connectivity
**Distance degree**	The sum of the corresponding row values in the distance matrix for each atom
**Eccentricity**	The greatest value in the corresponding row of the distance matrix for each atom
**Harary index**	Half-sum of the off-diagonal elements of the reciprocal molecular distance matrix of the molecule
**Hyper Wiener index**	A variant of the Wiener index
**Szeged index**	The Szeged index extends the Wiener index for cyclic graphs by counting the number of atoms on both sides of each bond (those atoms only which are nearer to the given side of the bond than to the other) and sum these counts
**Wiener index**	Average topological atom distance (half of the sum of all atom distances) in the molecule
**Wiener polarity**	Number of 3 bond length distances in the molecule
**Other Topological Descriptors**	
**Cyclomatic number**	Smallest number of bonds which must be removed so that no circuit remains. Also known as circuit rank
**Fragment count**	Number of fragments in the sketch
**Steric effect index**	Topological steric effect index (TSEI) of an atom calculated from the covalent radii values and topological distances. The steric Effect Index is related to the steric hindrance of the given atom
**Fsp3**	Number of sp^3 hybridized carbons divided by the total carbon count

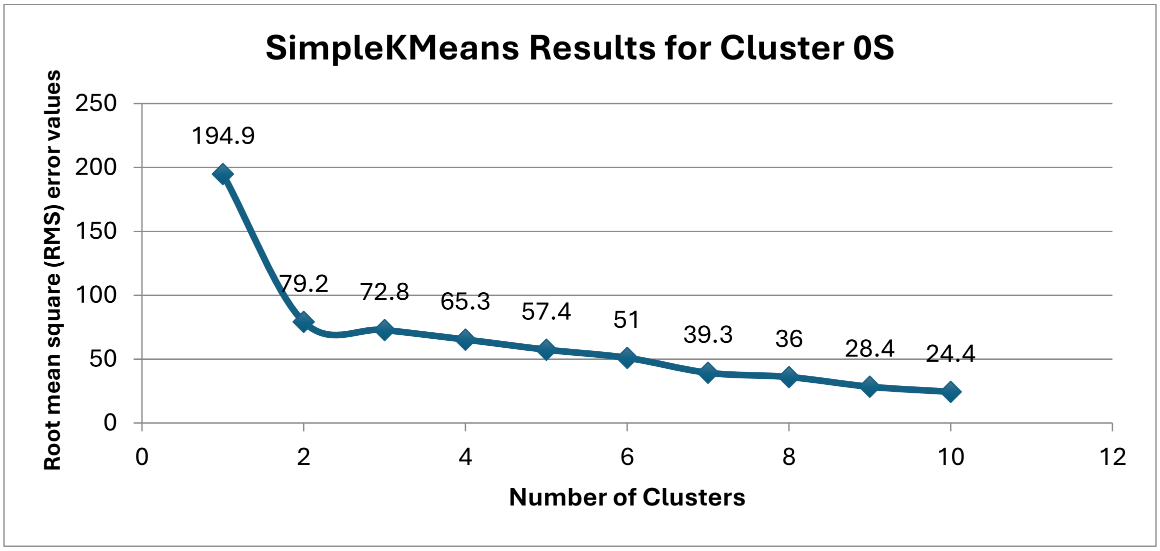
SimpleKMeans Results for Cluster 0S
Root mean square (RMS) error values
Number of Clusters
194.9
79.2
72.8
65.3
57.4
51
39.3
36
28.4
24.4

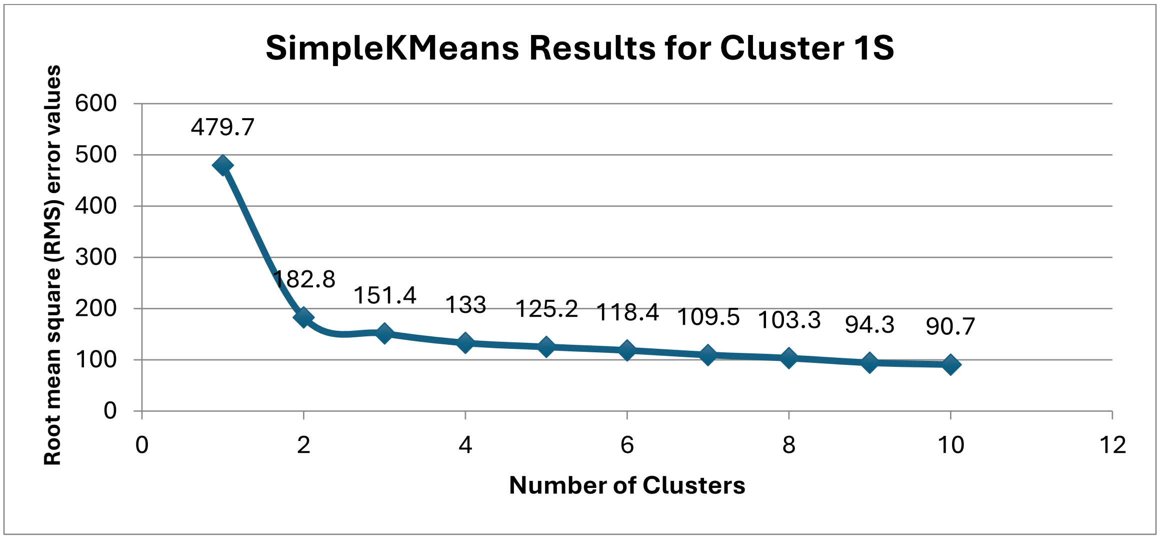
SimpleKMeans Results for Cluster 1S
Root mean square (RMS) error values
Number of Clusters
479.7
182.8
151.4
133
125.2
118.4
109.5
103.3
94.3
90.7

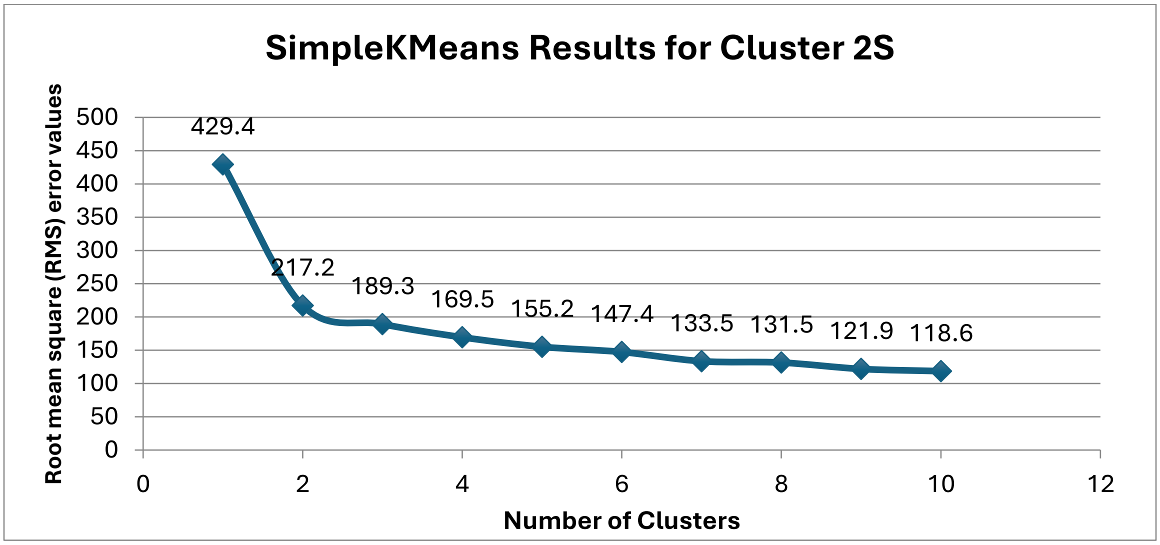
SimpleKMeans Results for Cluster 2S
Root mean square (RMS) error values
Number of Clusters
429.4
217.2
189.3
169.5
155.2
147.4
133.5
131.5
121.9
118.6

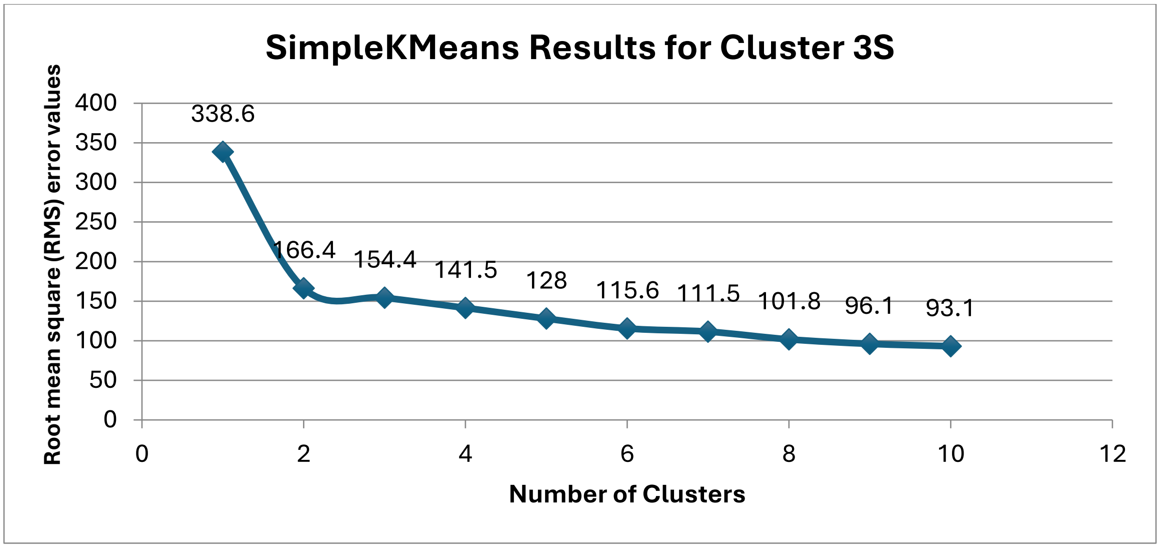
SimpleKMeans Results for Cluster 3S
Root mean square (RMS) error values
400
350
300
250
200
150
100
50
0
338.6
166.4
154.4
141.5
128
115.6
111.5
101.8
96.1
93.1
0
2
4
6
8
10
12
Number of Clusters

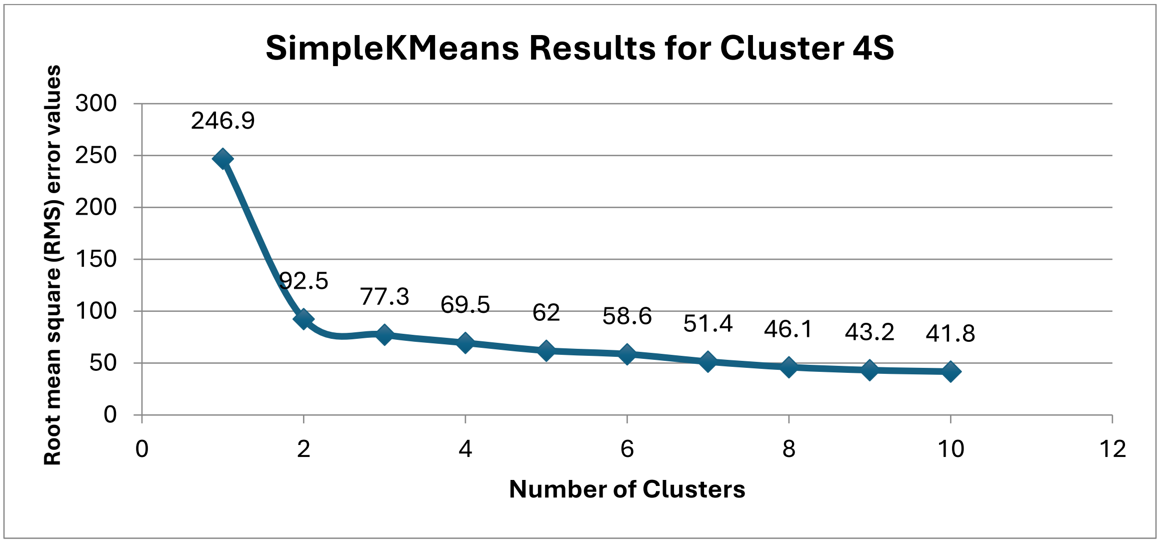
SimpleKMeans Results for Cluster 4S
Root mean square (RMS) error values
300
250
200
150
100
50
0
246.9
92.5
77.3
69.5
62
58.6
51.4
46.1
43.2
41.8
0
2
4
6
8
10
12
Number of Clusters

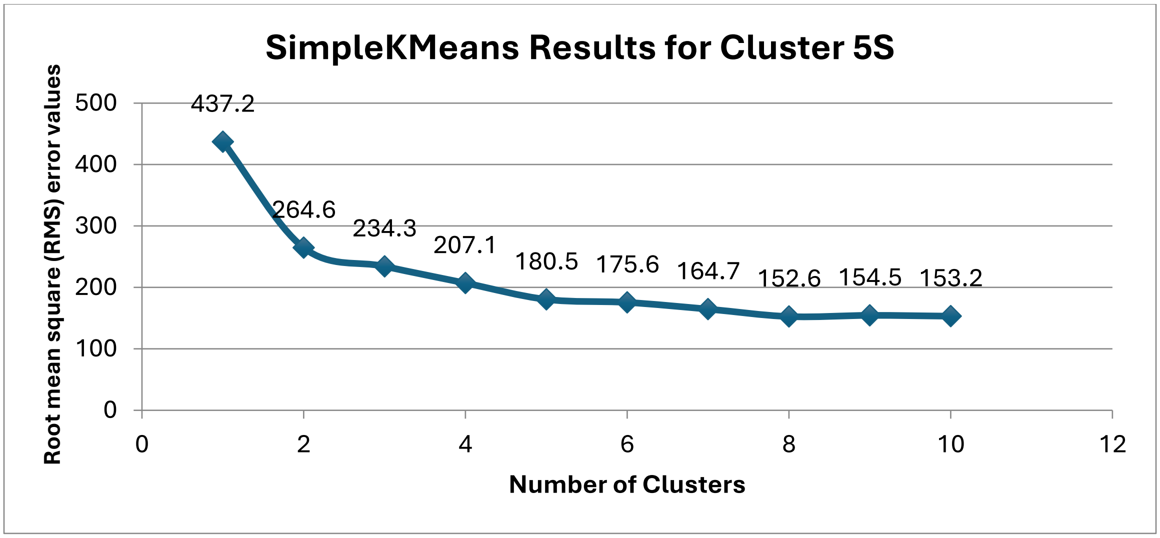
SimpleKMeans Results for Cluster 5S
Root mean square (RMS) error values
500
400
300
200
100
0
437.2
264.6
234.3
207.1
180.5
175.6
164.7
152.6
154.5
153.2
0
2
4
6
8
10
12
Number of Clusters

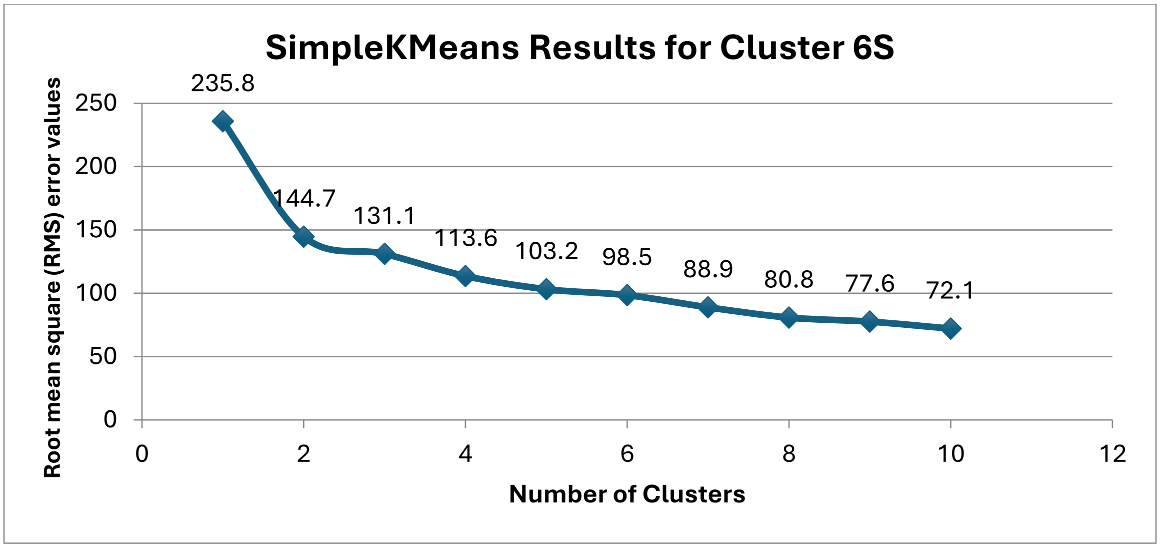
SimpleKMeans Results for Cluster 6S
Root mean square (RMS) error values
Number of Clusters
235.8
144.7
131.1
113.6
103.2
98.5
88.9
80.8
77.6
72.1
0
50
100
150
200
250
0
2
4
6
8
10
12

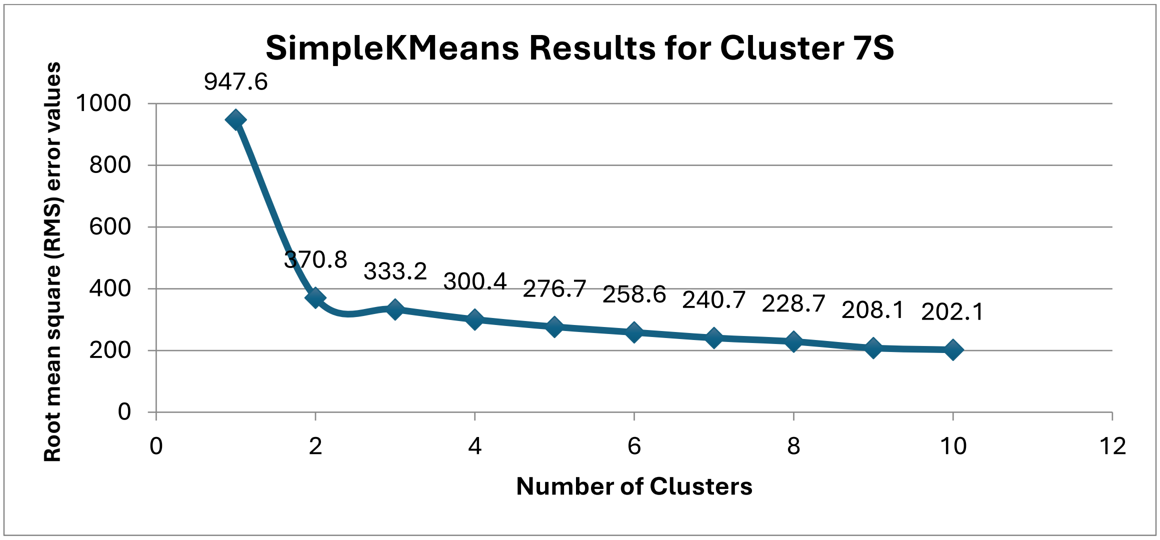
SimpleKMeans Results for Cluster 7S
Root mean square (RMS) error values
Number of Clusters
947.6
370.8
333.2
300.4
276.7
258.6
240.7
228.7
208.1
202.1
0
200
400
600
800
1000
0
2
4
6
8
10
12

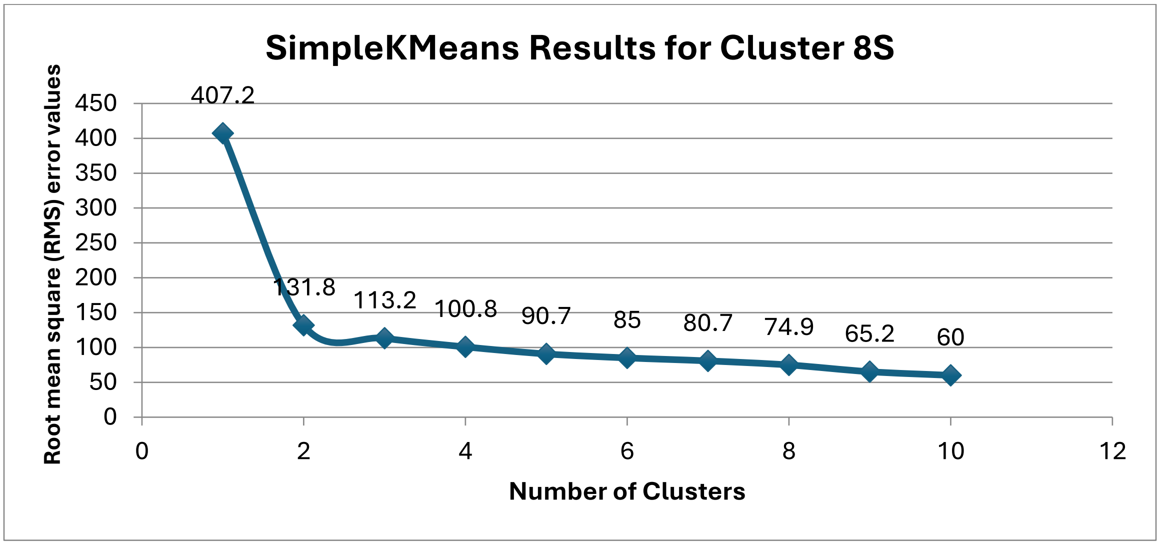
SimpleKMeans Results for Cluster 8S
Root mean square (RMS) error values
Number of Clusters
407.2
131.8
113.2
100.8
90.7
85
80.7
74.9
65.2
60
0
50
100
150
200
250
300
350
400
450
0
2
4
6
8
10
12

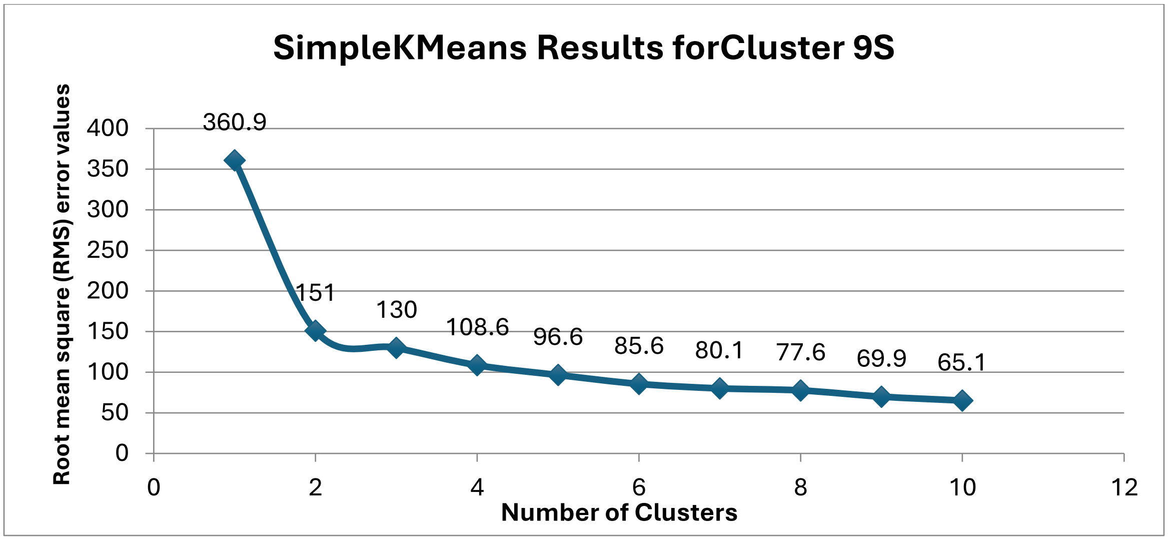
SimpleKMeans Results forCluster 9S
Root mean square (RMS) error values
400
350
300
250
200
150
100
50
0
360.9
151
130
108.6
96.6
85.6
80.1
77.6
69.9
65.1
0
2
4
6
8
10
12
Number of Clusters

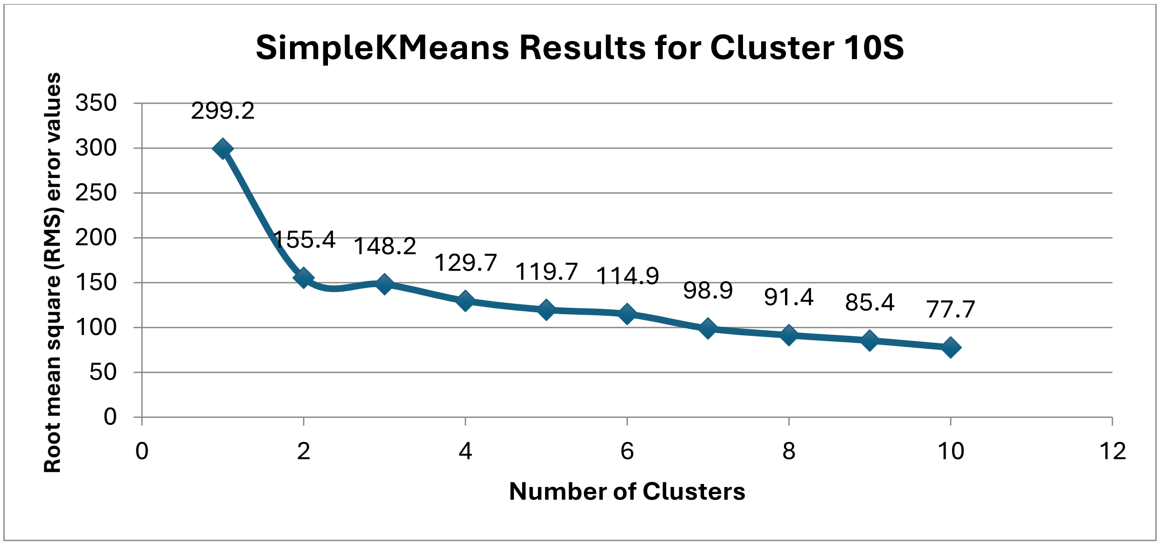
SimpleKMeans Results for Cluster 10S
Root mean square (RMS) error values
350
300
250
200
150
100
50
0
299.2
155.4
148.2
129.7
119.7
114.9
98.9
91.4
85.4
77.7
0
2
4
6
8
10
12
Number of Clusters

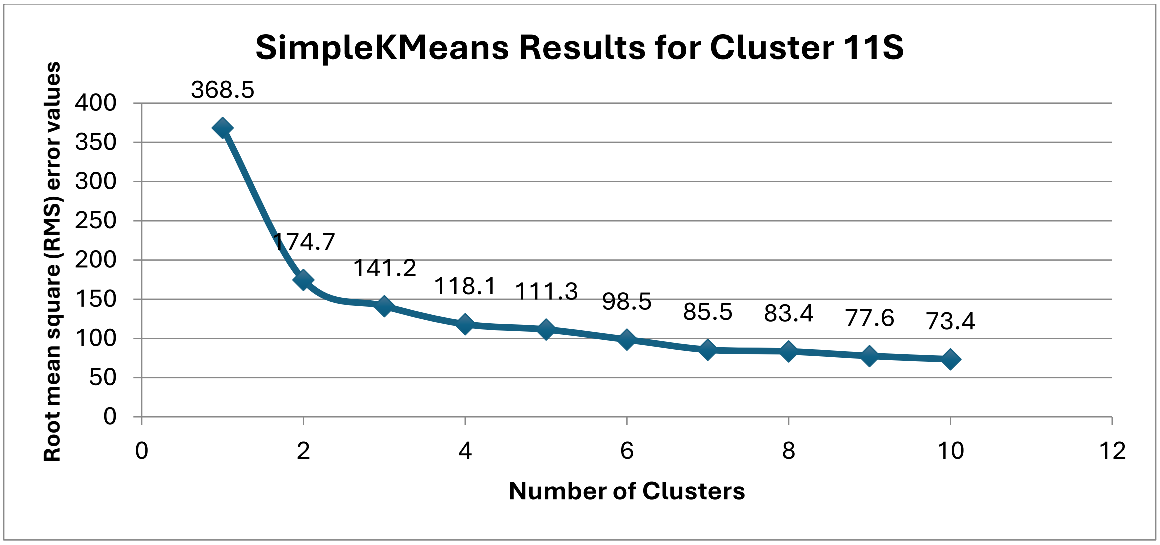
SimpleKMeans Results for Cluster 11S
Root mean square (RMS) error values
400
350
300
250
200
150
100
50
0
368.5
174.7
141.2
118.1
111.3
98.5
85.5
83.4
77.6
73.4
0
2
4
6
8
10
12
Number of Clusters

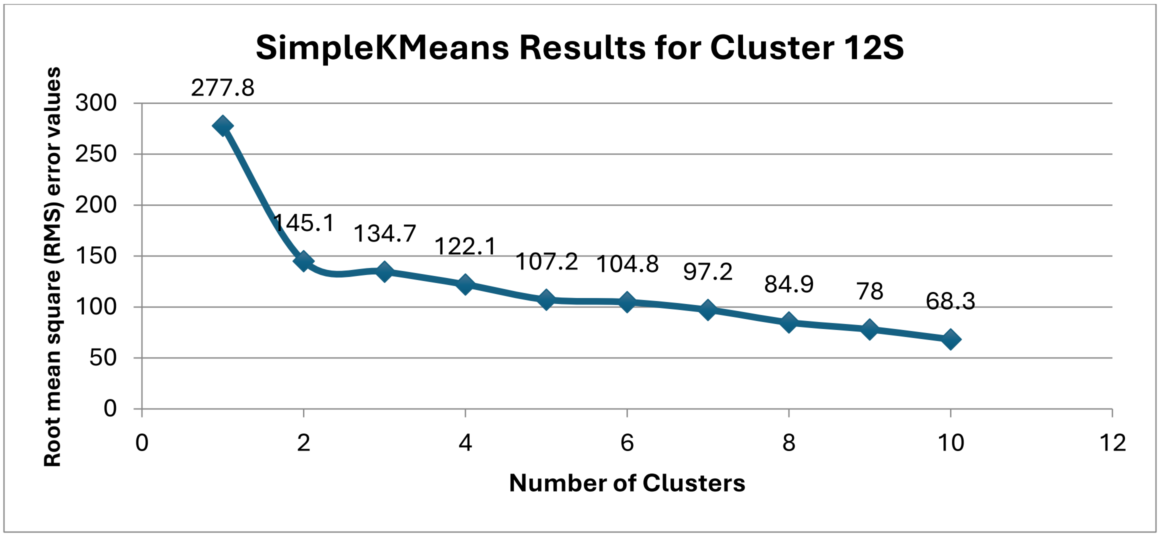
SimpleKMeans Results for Cluster 12S
Root mean square (RMS) error values
300
250
200
150
100
50
0
277.8
145.1
134.7
122.1
107.2
104.8
97.2
84.9
78
68.3
0
2
4
6
8
10
12
Number of Clusters

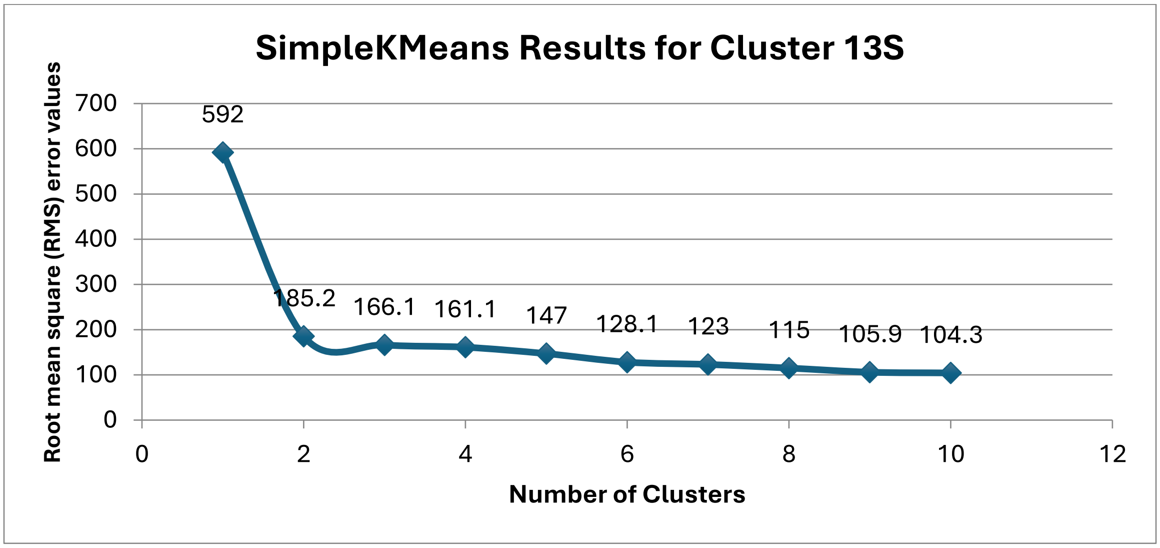
SimpleKMeans Results for Cluster 13S
Root mean square (RMS) error values
700
600
500
400
300
200
100
0
592
185.2
166.1
161.1
147
128.1
123
115
105.9
104.3
0
2
4
6
8
10
12
Number of Clusters

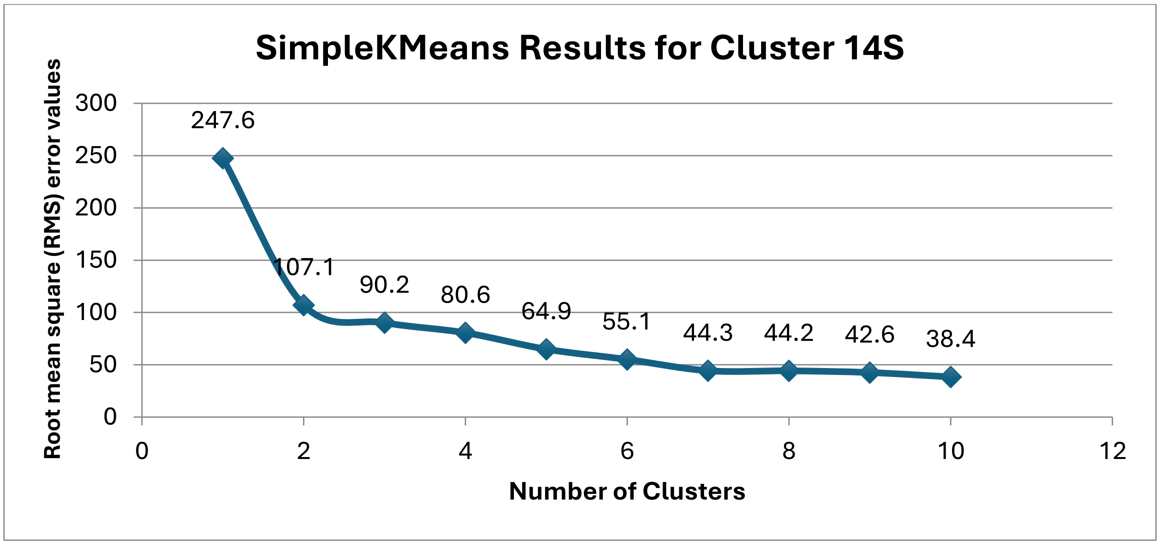
SimpleKMeans Results for Cluster 14S
Root mean square (RMS) error values
300
250
200
150
100
50
0
247.6
107.1
90.2
80.6
64.9
55.1
44.3
44.2
42.6
38.4
0
2
4
6
8
10
12
Number of Clusters

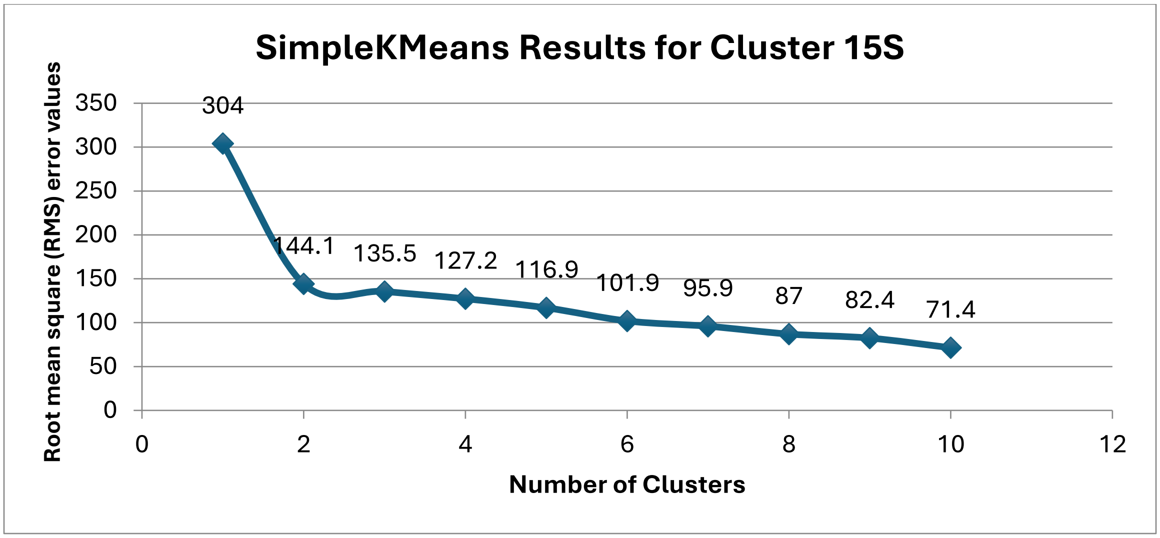
SimpleKMeans Results for Cluster 15S
Root mean square (RMS) error values
Number of Clusters
304
144.1
135.5
127.2
116.9
101.9
95.9
87
82.4
71.4
0
50
100
150
200
250
300
350
0
2
4
6
8
10
12

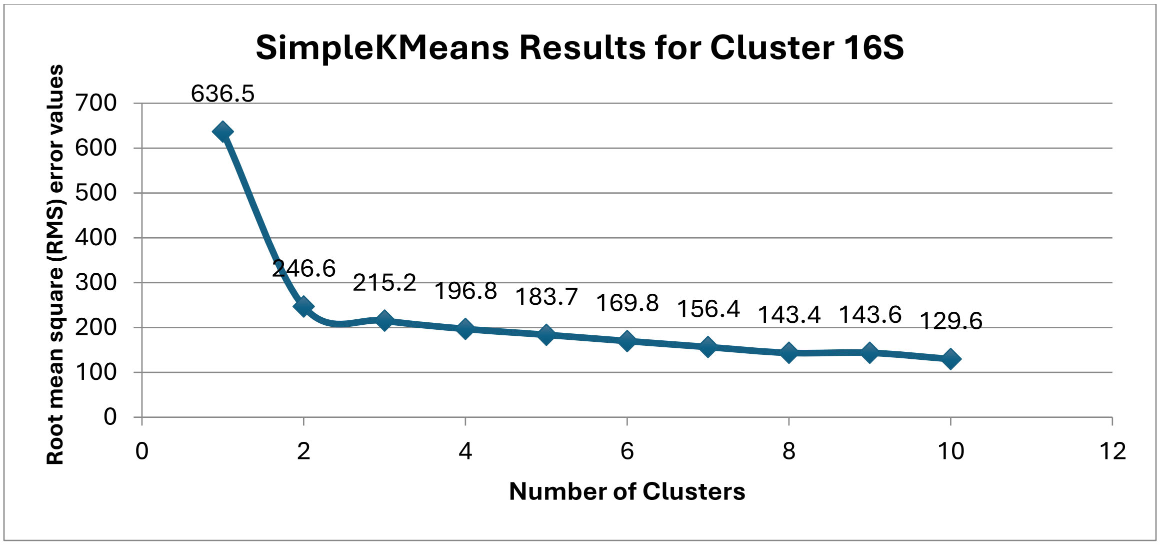
SimpleKMeans Results for Cluster 16S
Root mean square (RMS) error values
Number of Clusters
636.5
246.6
215.2
196.8
183.7
169.8
156.4
143.4
143.6
129.6
0
100
200
300
400
500
600
700
0
2
4
6
8
10
12

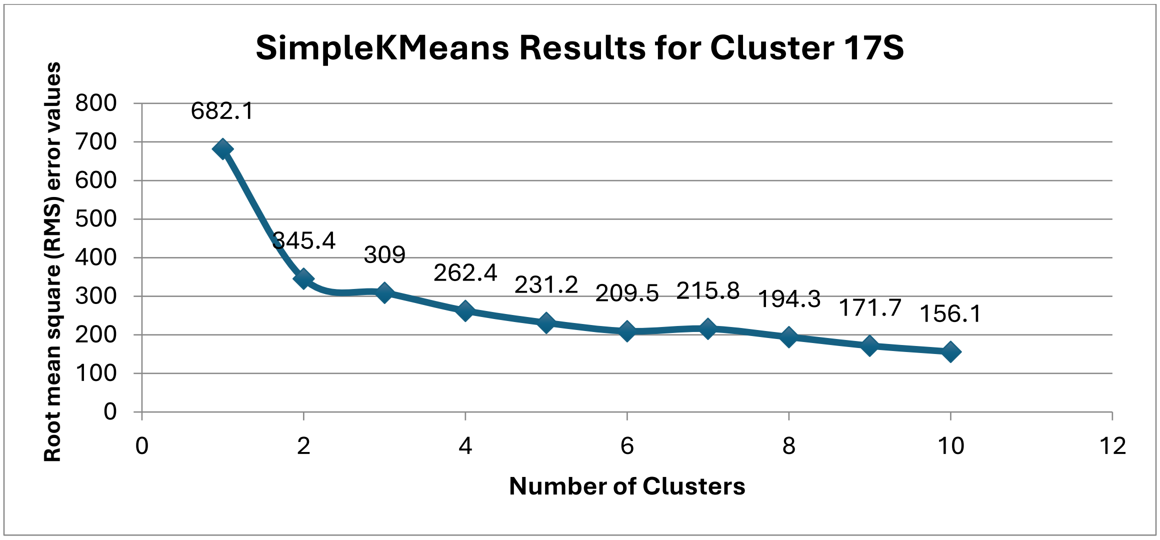
SimpleKMeans Results for Cluster 17S
Root mean square (RMS) error values
Number of Clusters
682.1
345.4
309
262.4
231.2
209.5
215.8
194.3
171.7
156.1
0
100
200
300
400
500
600
700
800
0
2
4
6
8
10
12

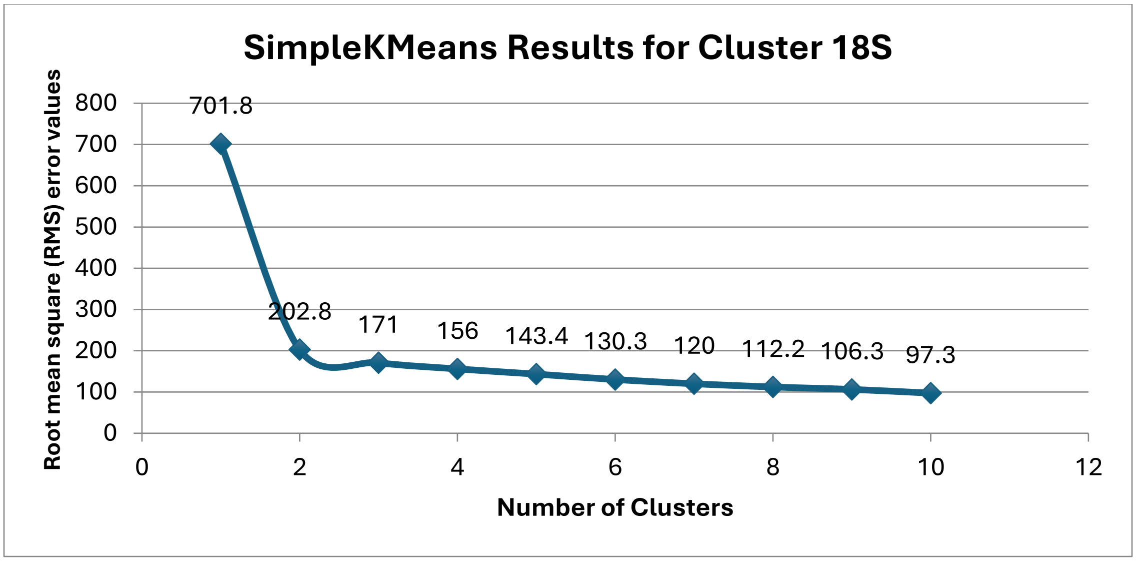
SimpleKMeans Results for Cluster 18S
Root mean square (RMS) error values
Number of Clusters
701.8
202.8
171
156
143.4
130.3
120
112.2
106.3
97.3

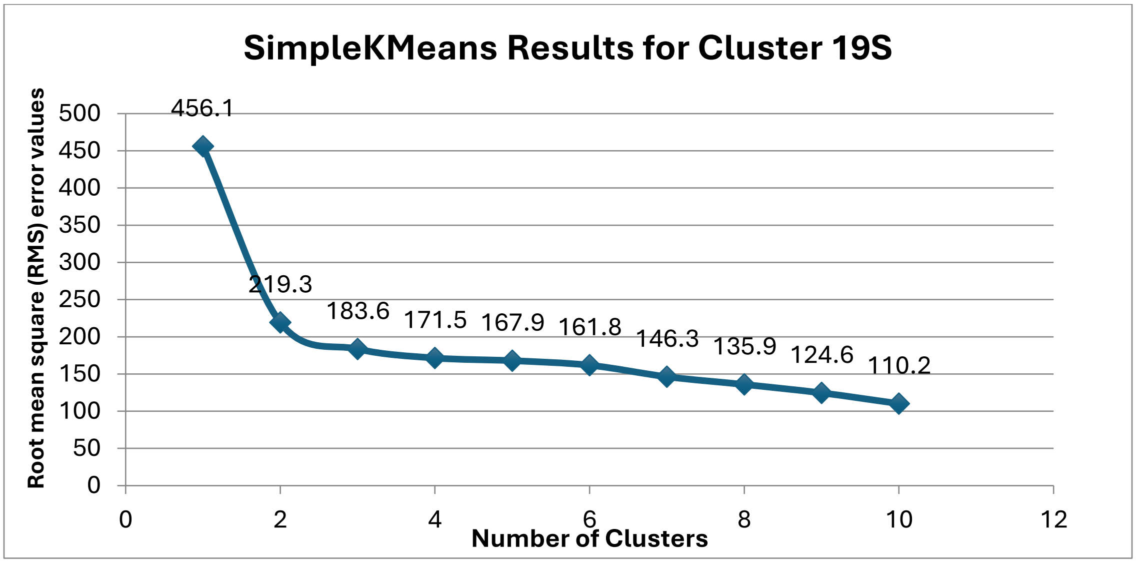
SimpleKMeans Results for Cluster 19S
Root mean square (RMS) error values
Number of Clusters
456.1
219.3
183.6
171.5
167.9
161.8
146.3
135.9
124.6
110.2

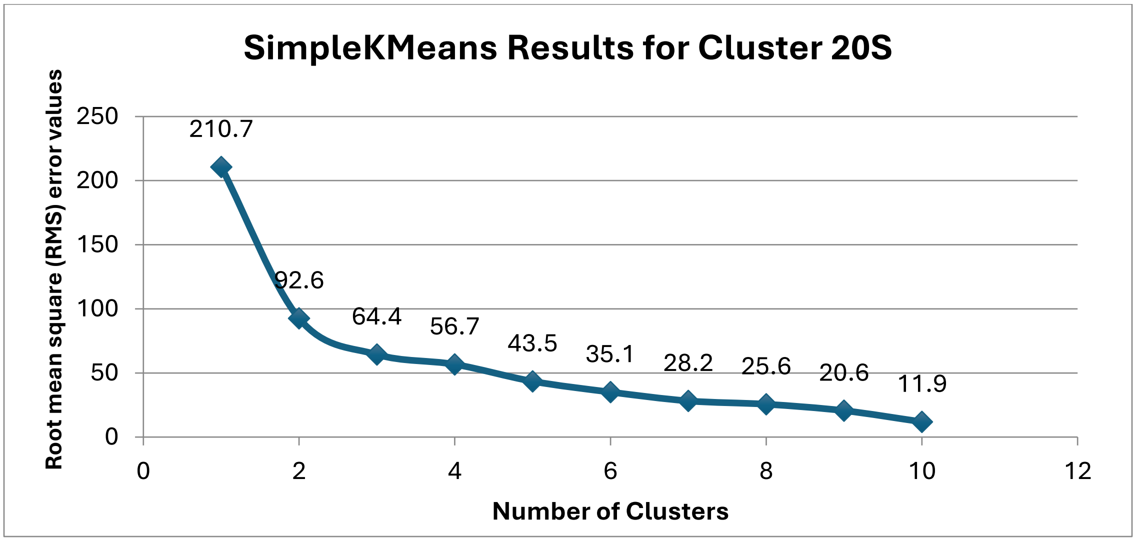
SimpleKMeans Results for Cluster 20S
Root mean square (RMS) error values
Number of Clusters
210.7
92.6
64.4
56.7
43.5
35.1
28.2
25.6
20.6
11.9

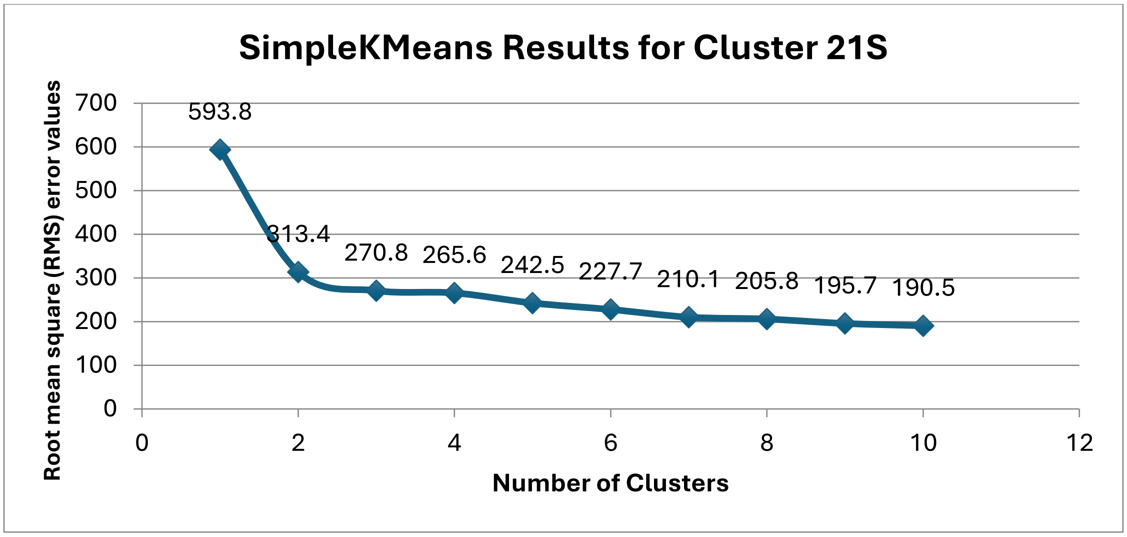
SimpleKMeans Results for Cluster 21S
Root mean square (RMS) error values
Number of Clusters
0
100
200
300
400
500
600
700
0
2
4
6
8
10
12
593.8
313.4
270.8
265.6
242.5
227.7
210.1
205.8
195.7
190.5

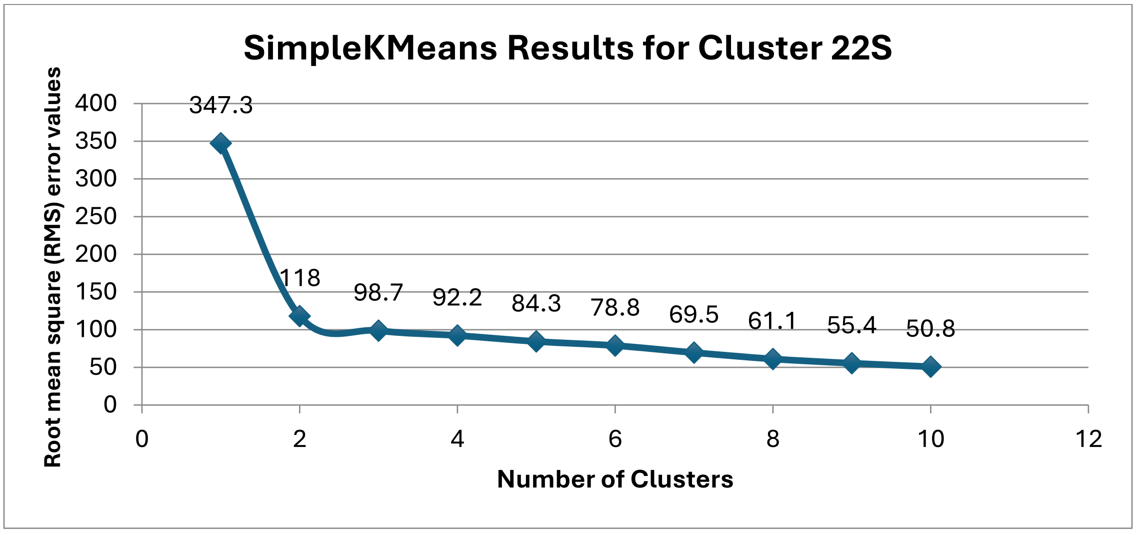
SimpleKMeans Results for Cluster 22S
Root mean square (RMS) error values
Number of Clusters
0
50
100
150
200
250
300
350
400
0
2
4
6
8
10
12
347.3
118
98.7
92.2
84.3
78.8
69.5
61.1
55.4
50.8

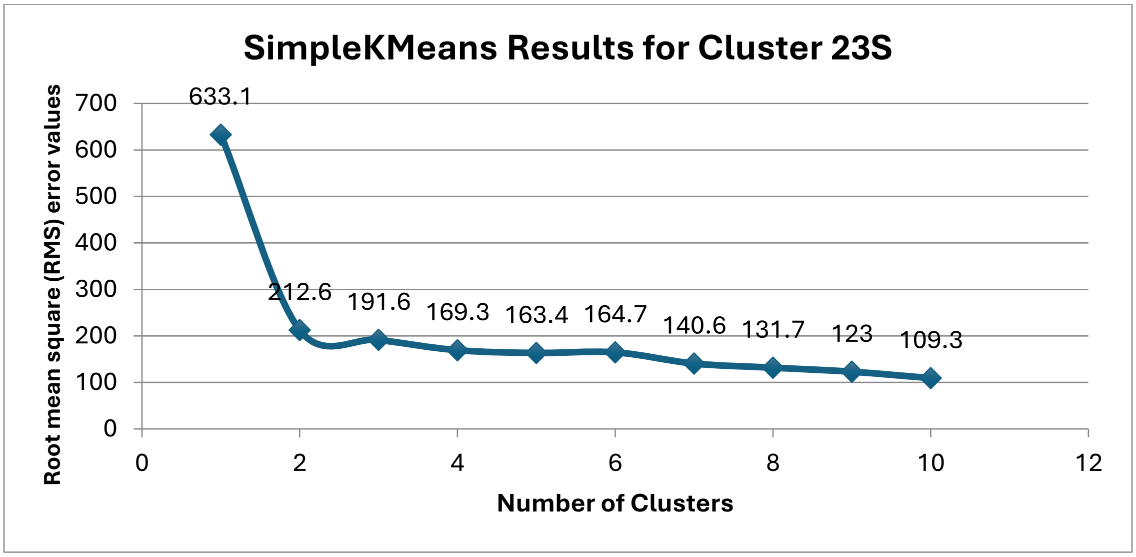
SimpleKMeans Results for Cluster 23S
Root mean square (RMS) error values
Number of Clusters
0
100
200
300
400
500
600
700
0
2
4
6
8
10
12
633.1
212.6
191.6
169.3
163.4
164.7
140.6
131.7
123
109.3

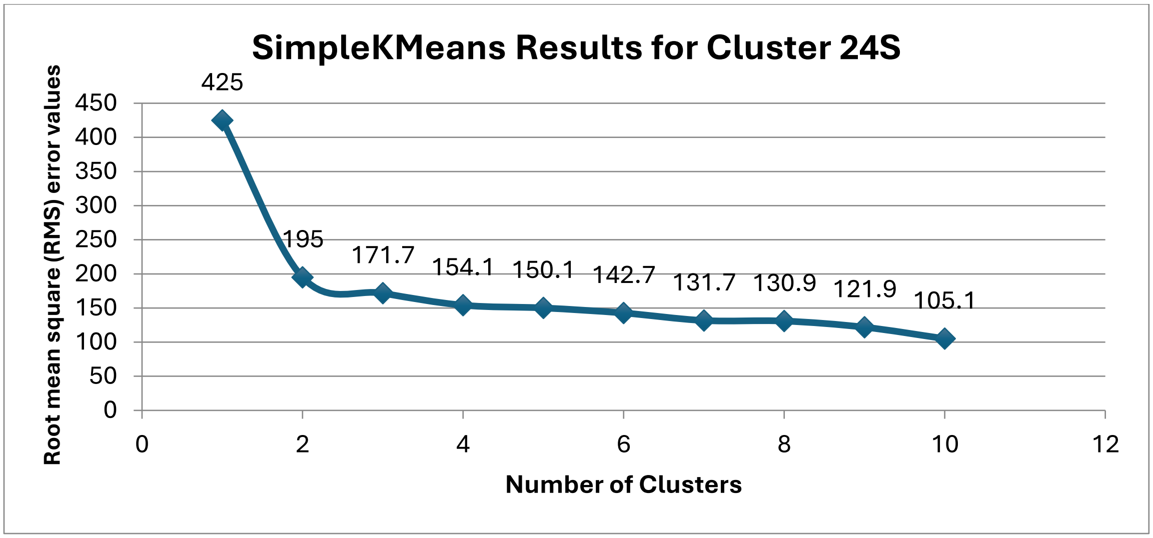

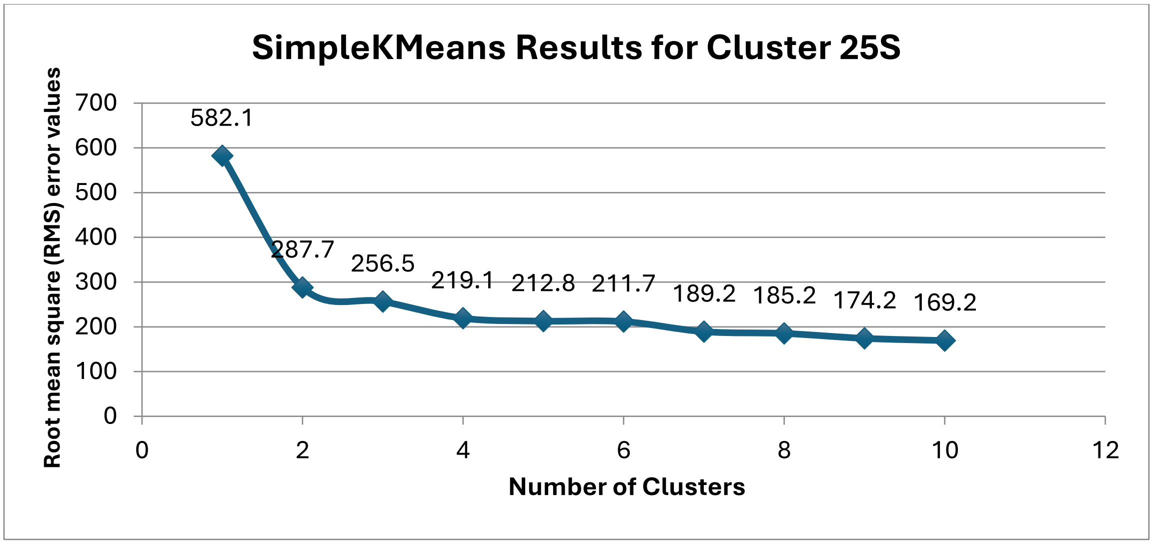

Supplementary Figure 1. Plots showing the average root mean square (RMS) error values from the SimpleKMeans clustering algorithm for each cluster in the S model. The x-axis at the elbow of each graph indicates the optimal number of subclusters each cluster should be grouped into based on the algorithm.

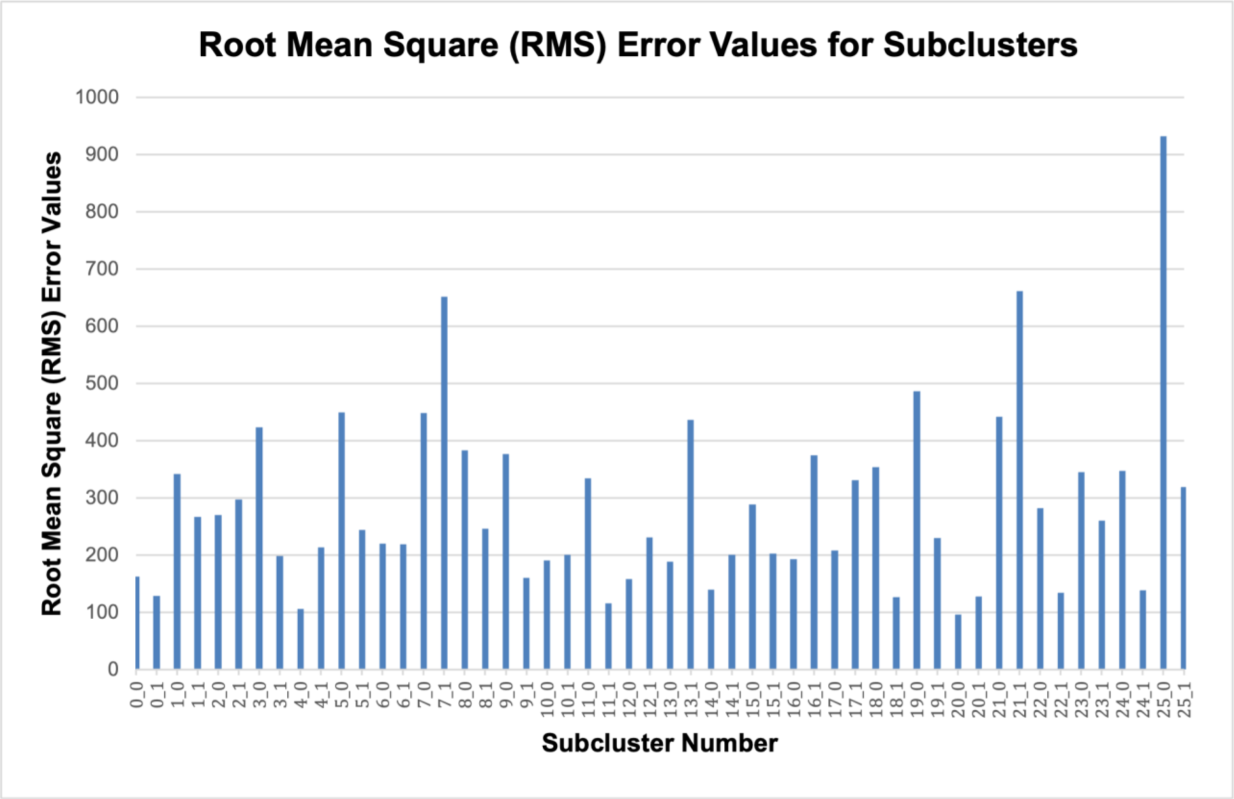

Supplementary Figure 2: Root mean square (RMS) error values for all subclusters within each screening (S) model cluster from the SimpleKMeans algorithm. Lower RMS error values indicate lower variance within the subcluster, and higher RMS values indicated higher variance within the subcluster. Lower RMS error values indicate a tighter cluster of compounds with greater shared structural properties. The x-axis indicates the subcluster number in the S model. The y-axis indicates the RMS error value for each subcluster. Lower bars correlate with lower RMS error values and higher bars correlate with higher RMS error values.

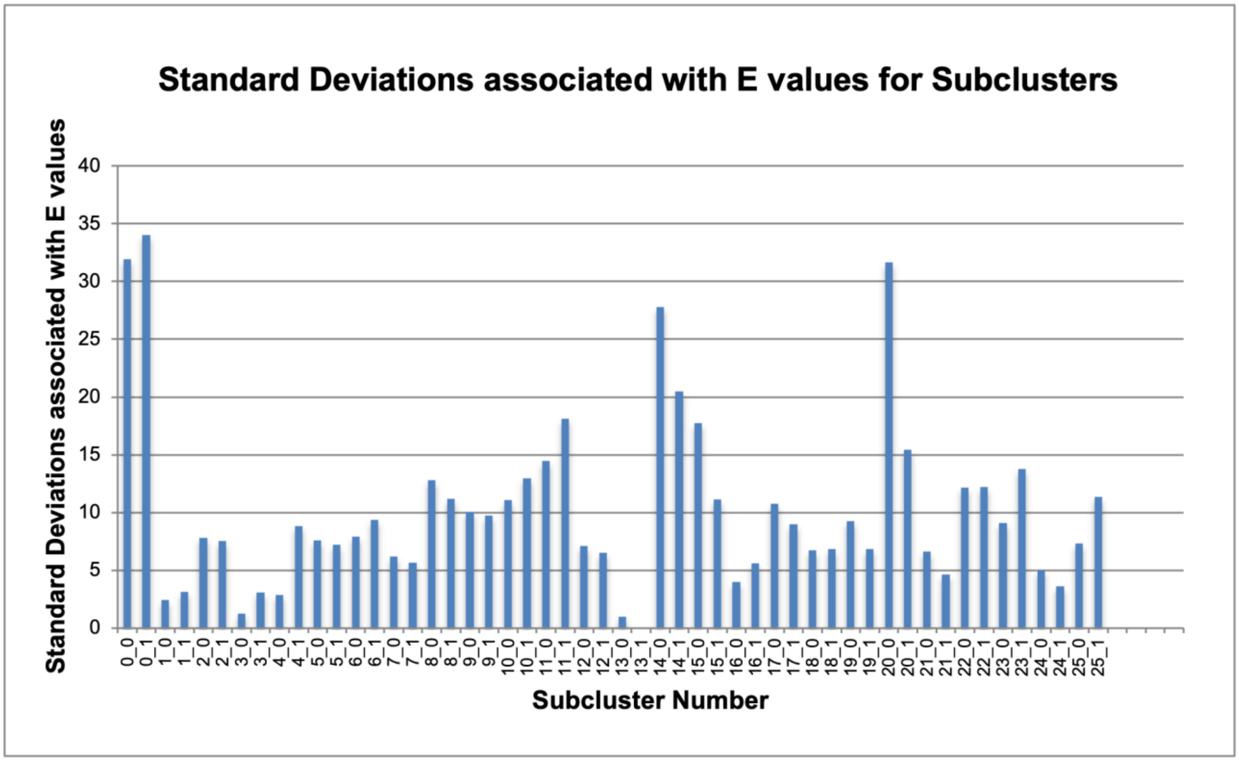

Supplementary Figure 3: Standard deviations associated with E values for all subclusters within each screening (S) model cluster. The E value is an effectiveness value that measures the capability of a compound to inhibit *ATXN2*. The standard deviations associated with the E values give insight into compounds that have similar inhibition properties. Lower standard deviations indicate subclusters with shared inhibition properties whereas higher standard deviations indicate subclusters with greater variability in inhibition properties. The x-axis indicates the subcluster number from the S model. The y-axis indicates the standard deviation associated with the E value of each subcluster. Lower bars correlate with lower standard deviations and higher bars correlate with higher standard deviations.

Supplementary Table 2: Root mean square (RMS) error values as well as mean E values and standard deviations associated with mean E values for each subcluster from the screening only (S) clusters.

Subcluster	RMS Error Values	Mean E values	Standard Deviation of Mean E values
0_0	162.7	-76.1099	31.9296
0_1	128.4	-71.1385	34.0093
1_0	341.5	-0.3417	2.4462
1_1	266.4	-0.2712	3.1468
2_0	270.1	13.1879	7.8149
2_1	297.5	11.5466	7.5264
3_0	423	0.3862	1.2636
3_1	198.1	-0.8813	3.053
4_0	105.5	84.5985	2.8878
4_1	213.5	71.8743	8.8049
5_0	449.4	-8.866	7.6131
5_1	243.7	-4.5633	7.192
6_0	220.3	48.6041	7.9192
6_1	218.8	51.9813	9.3518
7_0	449	13.1427	6.1695
7_1	652.3	12.5493	5.6589
8_0	383.2	-20.0815	12.7852
8_1	246.5	-19.8318	11.1971
9_0	376.9	41.3143	10.0688
9_1	160.2	39.6876	9.7412
10_0	190.5	7.7108	11.0966
10_1	200.8	5.8659	12.9816
11_0	334.1	-25.8527	14.4768
11_1	115.5	-25.5361	18.1234
12_0	158	2.9101	7.0983
12_1	231.2	0.1366	6.5223
13_0	188.7	0.3037	0.973
13_1	436.7	0.0047	0.0259
14_0	139.7	-31.3378	27.7847
14_1	200.9	-48.8171	20.4787
15_0	289	21.548	17.762
15_1	202.7	24.7152	11.1104
16_0	192.9	2.6824	4.0168
16_1	374.5	2.8971	5.609
17_0	208.7	6.9179	10.742
17_1	331	8.0632	8.962
18_0	354.3	4.7447	6.7502
18_1	126.7	3.103	6.8324
19_0	487.1	31.6249	9.2478
19_1	229.4	32.6096	6.8631
20_0	96.4	18.9833	31.6368

20_1	127.9	19.6004	15.4156
21_0	442.4	-0.2934	6.6112
21_1	661.3	0.2353	4.66
22_0	281.9	-19.4048	12.1412
22_1	134.2	-8.5677	12.2316
23_0	345.2	7.9384	9.0756
23_1	260.2	9.8405	13.7489
24_0	346.9	4.2945	4.9947
24_1	138.9	7.2688	3.6255
25_0	932.2	20.9117	7.3095
25_1	319.3	21.4289	11.3655

Blue indicates subclusters that contained compounds which were further examined visually since they had low RMS error values and low standard deviation associated with E values (subclusters 4_0 and 24_1).

A

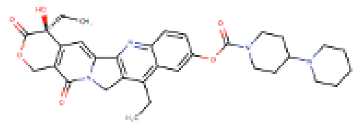

B

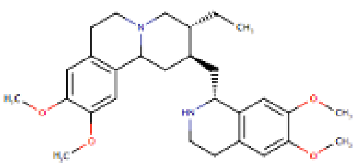

C

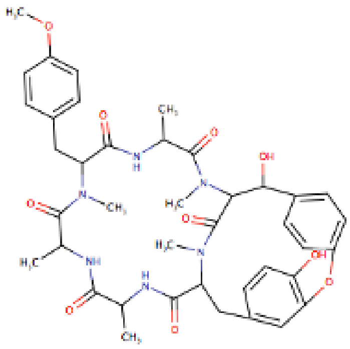

D

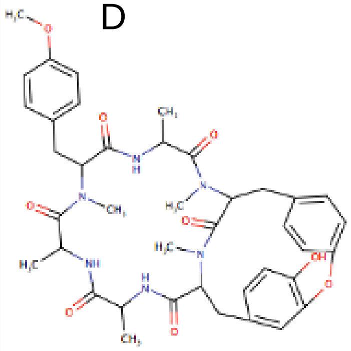

E

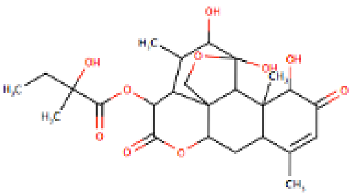

F

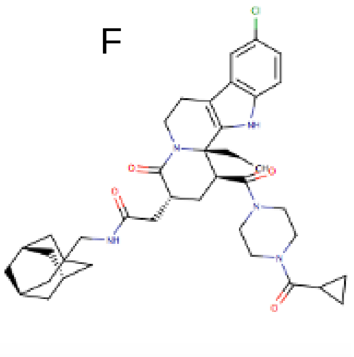

G

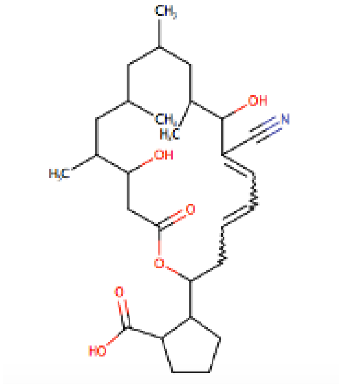

Supplementary Figure 4. Chemical structures of the seven weak *ATXN2* inhibiting compounds from subcluster 24_1.
